# Interoception and obesity: a systematic review and meta-analysis of the relationship between interoception and BMI

**DOI:** 10.1038/s41366-021-00950-y

**Published:** 2021-09-03

**Authors:** Eric Robinson, Georgia Foote, Jemma Smith, Suzanne Higgs, Andrew Jones

**Affiliations:** 1 Institute of Population Health Sciences, University of Liverpool, Liverpool, UK; 2 School of Psychology, University of Birmingham, Birmingham, UK

**Keywords:** Risk factors, Neuroscience

## Abstract

**Background:**

Interoception refers to the processes by which we sense, interpret and integrate signals originating from within the body. Deficits in interoception have been linked to higher BMI and may contribute to weight gain. However, there have been conflicting findings and it is not clear how higher BMI is associated with different facets of interoception, such as interoceptive accuracy (the ability to detect internal signals) and sensibility (the tendency to attend to internal signals).

**Methods:**

We conducted a systematic review and meta-analysis of studies that measured interoception and BMI. We examined relationships between interoception and BMI in children and adults separately and as a function of interoceptive facet and measure. In sensitivity analyses, we tested for evidence of publication bias and whether the results were consistent when studies with a high risk of bias were excluded.

**Results:**

A total of 87 articles were eligible for inclusion. In adults (121 effects, 10,425 participants), there was cross-sectional evidence of higher BMI being associated with overall deficits in interoception (*r* = −0.054, 95% CI: −0.084 to −0.025) and this was consistent across sensitivity analyses. There was no statistically significant evidence of moderation by interoceptive facet or measure, although there was some variability in effect size estimates based on interoceptive facet and measures. A smaller meta-analysis limited to studies that compared participants with normal weight vs. overweight/obesity indicated poorer interoception in participants with overweight/obesity (SMD = −0.39, 95% CI −0.60 to −0.18).

**Conclusions:**

In cross-sectional studies, deficits in interoception are associated with higher BMI. However, it remains unclear whether deficits in interoception contribute to or are a consequence of weight gain and obesity.

## Introduction

Interoception is a multi-faceted construct that relates to a range of processes that determine the extent to which we sense, interpret and integrate signals originating from within the body [1]. Interoceptive ‘accuracy’ is the ability to detect internal bodily signals [2], such as one’s own heartbeat or distension of the stomach after eating. In line with Murphy’s proposed factorial structure of interoception and its measurement [3], interoceptive accuracy can be measured through objectively measured task performance and perceived accuracy can be measured through participant self-report. For example, there are individual differences in the ability to track the number of times one’s own heartbeats during a time period; the heartbeat counting task [4]. Likewise, interoceptive accuracy has also been measured through examination of the extent to which people can identify whether presented heartbeat intervals are congruent or incongruent with their own current heartbeat; the heartbeat discrimination task [5]. A different facet of interoception is interoceptive ‘sensibility’; the tendency to attend or attempt to ‘listen’ to internal body signals [2]. Although interoceptive accuracy and sensibility have received the most empirical attention, recent theoretical models proposed by Garfinkel and Khalsa outline a number of other facets of interoception, including ‘awareness’; meta-cognitive awareness of interoceptive accuracy, ‘prediction error’; level of concordance between interoceptive sensibility and interoceptive accuracy [1] and ‘discrimination’; sensing of localised sensations to a specific bodily area and the ability to discriminate from other sensations [2]. Recent theoretical models and empirical work [6] also outline the importance of distinguishing between different type of sensory modality (e.g., cardiac, pain, gastric) when examining interoceptive processes.

A growing body of research suggests that interoceptive processes may play an important role in shaping health [7]. Because interoceptive sensations are thought to be a basis of emotional feelings [8], impaired or exaggerated interoception may interfere with emotion interpretation or regulation and therefore increase the risk of developing mental health conditions [9]. In line with this reasoning, compared to healthy controls altered interoception has been identified in patients with depression, anxiety and eating disorders [10–12].

Interoception may also play an important role in explaining individual differences in overweight (BMI ≥ 25–29.9) and obesity (BMI ≥ 30). In the modern-day food environment, there are a large number of ‘external’ cues on eating behaviour [13], as well as the wide availability of calorie-dense food [14]. However, how much we eat is also influenced by internal signals, including expansion of the stomach, circulating appetite hormones and accompanying changes in general autonomic arousal [15]. Deficits in interoception could therefore result in internal appetite signals that encourage satiety being less strongly weighted into eating and food-related decision-making [16], which in turn would be hypothesised to increase the risk of weight gain in the modern-day ‘obesogenic’ food environment. In line with this proposal, participants with lower self-reported interoceptive accuracy are less likely to report eating in response to internal and satiety signals [17, 18]. Interoceptive processes may also contribute to the development of obesity through emotional regulation. A well-recognised contributor to weight gain is eating in response to negative emotions [19, 20]. As deficits in interoception may interfere with emotion regulation, such deficits may indirectly increase susceptibility to emotional overeating and subsequent weight gain [21]. Supporting this hypothesis, studies have linked interoceptive processes to trait emotional eating and binge eating [16, 22].

Although deficits in interoceptive processes are a risk factor for disordered eating patterns [16], the role that interoceptive processes have in the development and maintenance of obesity is less clear. Studies in which interoceptive accuracy has been measured using the heartbeat counting task and heartbeat discrimination task have found higher BMI to be associated with deficits in interoceptive accuracy [23, 24]. However, recent studies have not consistently found significant associations between interoception and BMI [21, 25, 26]. For example, both Young et al. [21] and Mata et al. [25] found no association between BMI and objective measures of cardiac interoceptive accuracy, whereas Robinson et al. [27] found a significant negative association between BMI and self-reported perceived interoceptive accuracy. Likewise, two recent studies adopting different measures of interoceptive sensibility found no significant association between sensibility and BMI [26, 27]. These mixed findings may be in part explained by a range of factors, including the use of different interoception measurement tools. For example, there is growing awareness that the heartbeat counting task is unlikely to be a valid measure of interoceptive accuracy [28]. Furthermore, it is unclear how different facets of interoception relate to obesity and whether there is evidence that deficits in interoception predict weight gain and the development of obesity [29].

The aim of the present research was to systematically review and meta-analyse all available studies that allow for quantification of the relationship between interoception and higher BMI. Because there are multiple facets of interoception (e.g., interoceptive accuracy, sensibility) and a range of measurement tools used to measure interoceptive processes, a secondary aim was to examine whether any relationship between interoception and BMI was moderated by interoceptive facet type and measure. To understand potential temporal relations between interoception and BMI (i.e., are deficits in interoception a cause or consequences of higher BMI?), we aimed to synthesise results in children and adults separately, as well as examining cross-sectional studies (concurrent measurement of interoception and BMI) and prospective studies (interoception predicting future BMI) separately.

## Methods

### Study selection and eligibility criteria

#### Participants

Studies of adults and children were eligible. Studies that only sampled participants with a medical/psychiatric condition (e.g., eating disorder, diabetes) were ineligible due to concerns over generalisability. If a study recruiting a clinical sample also recruited an otherwise healthy control/comparison group, the data from the healthy group was eligible for inclusion.

#### Interoception measures

To be eligible for inclusion studies were required to have measured at least one of the following facets of interoception, as outlined by [3]; *accuracy*; the ability to detect interoceptive signals, *sensibility*; the tendency to focus on interoceptive signals, *awareness*; meta-cognitive awareness of interoceptive accuracy, *prediction error*; level of concordance between interoceptive sensibility and interoceptive accuracy [30]. Both self-reported (‘perceived accuracy’) and objective task performance measures of interoception were eligible. In line with Murphy’s factorial model [3], examples of eligible measures for accuracy were as follows; the heartbeat discrimination [24] and counting tasks [23], the interoceptive accuracy scale [3] and self-reported confidence in accuracy (e.g., measured during an objective task). Examples of eligible measures for sensibility were as follows; Porge’s body perception questionnaire [31], the listening subscale of the Multidimensional Assessment of Interoceptive Awareness (MAIA) scale [32]. If studies did not directly measure interoception they were not eligible. For example, the body awareness questionnaire [33] was ineligible because items relate to self-reported information on sleep-waking cycles as well as interoceptive accuracy. For more detailed information interoception measures and eligibility criteria, see Online Supplementary Materials. In our pre-registered review protocol, we classified the widely used interoceptive awareness (IA) subscale of the Eating Disorders Inventory (EDI) as a measure of interoceptive accuracy. However, a number of scale items are unlikely to directly measure interoceptive accuracy and it is therefore better categorised as a non-specific measure of interoception. Given that a large number of studies have examined the relationship between BMI and the EDI-IA, studies using this measure were retained for use and classed as ‘other’ for the purpose of meta-analysis.

#### Comparisons

We included studies that used a group-comparison design (e.g., participants with normal weight vs. participant with overweight/obesity) and studies that examined the correlation between interoception and BMI.

#### Outcomes

During scoping, we found that BMI was used to characterise heavier body weight (e.g., no studies found during scoping used alternative indices, such as waist circumference), therefore for comparative purposes only studies that collected BMI data were eligible. Both self-reported BMI (i.e. calculated from self-reported weight and height) and researcher measured BMI were eligible.

#### Study designs

We excluded studies in which sampling was purposefully restricted to one weight status category (e.g., recruitment of participants only with a BMI in the normal weight range) because the lack of variability in BMI would not allow for a test of the association of interoception and higher BMI. Studies that only reported data on interoception after an experimental manipulation or intervention designed to alter interoception were ineligible. However, if data on baseline (pre-manipulation) interoception were reported the study was eligible. As our primary interest was in the potential impact that interoception has on higher BMI, cross-sectional studies (concurrent measurement) and prospective studies that examined the relation between baseline interoception and future weight change were eligible, whereas studies examining the impact of body weight on changes in interoception were not eligible.

### Article identification strategy

We searched PsycINFO, PubMed and SCOPUS from 1990 onwards. Before 1990, there was little systematic research using widely adopted and standardised measures of interoception and we reasoned that including studies prior to this date may not provide an accurate characterisation of the modern-day relation between interoception and BMI (see online supplementary materials for search terms used). During scoping searches, we noted that a number of eligible articles made no reference to BMI/body weight in the title or abstract, seemingly because although BMI was measured it was not a primary measure of interest nor analysed in relation to interoception. Hence, we reasoned that several articles would not be detectable using electronic searches alone. Therefore, to further increase our ability to identify as many eligible articles as possible, we used a snowballing approach by searching the reference lists of all eligible articles and examining forward citations (in Google Scholar). We also identified key literature reviews on interoception and health and searched reference lists of these articles. To identify grey literature and unpublished manuscripts, we searched the Open Science Framework preprint archive (which covers 30 other preprint archives, including PsychArxiv and Nutrixiv). Studies were not excluded on the basis of language (e.g., non-English language articles were eligible). Two authors independently screened and judged the eligibility of articles identified through electronic searches. A single author identified potentially eligible articles using the snowballing and grey literature methods described above, and all potentially eligible articles were then included/excluded after verification by a second independent author.

### Data extraction and missing information

Two authors independently extracted the following information: bibliographic information (authors, year of publication), summary information on participant age, gender, BMI of sample and country in which the research was conducted, measure of BMI used (self-report vs. measured BMI), any exclusion criteria used in recruitment, information on all eligible measure(s) of interoception and number of trials used in objective interoception task measures, classification of interoception measure type (self-reported, objective, mixed) and facet (e.g., accuracy, sensibility), number of participants in interoception-BMI analysis, study design (correlational vs. group-comparison, cross-sectional vs. prospective), results of statistical analyses (e.g., effect size) examining relationship between interoception–BMI and risk of bias evaluations (see below). If a study did not report the association between interoception and BMI (or the number of participants), we contacted the authors and requested this information as a Pearson’s *r*.

### Methodological quality and risk of bias

We consulted guidance for assessing bias [34, 35] and developed our own list of methodological/risk of bias indicators for studies examining the relationship between interoception and BMI. The list items included whether the study; (i) accounted for potential confounding effects of psychiatric conditions when examining the relation between interoception and BMI (e.g., screened and excluded participants with a psychiatric condition), (ii) had sufficient participant variability in BMI (e.g., ≥25% of participants with a BMI > 25), (iii) accounted for underweight participants when examining the linear relation between interoception and BMI (e.g., excluded underweight participants from analyses), (iv) had a very small sample size (e.g., *n* < 30 for correlational studies), (v) used non-validated/standardised methods to interoception (e.g., questionnaire without psychometric validation), (vi) was pre-registered. For full details and justification of each risk of bias indicators, see Online Supplementary Materials Document.

### Analysis approach

#### Primary analyses

See https://osf.io/wfb96/ for pre-registered analysis plan. We converted all extracted effect sizes into the most commonly reported effect size (*r*). For analyses we planned to perform Fisher’s z transformation (*z* = 0.5*ln((1 + *r*)/ln(1 − *r*))) on *r* values, to improve normality. Variance of Fisher’s *Z* was calculated as 1/(*n* − 3). Note that we converted Fisher’s *Z* back to *r* values in text for summary statistics, whereas Fisher’s *Z* transformed values are reported in figures. To account for duplicity, when the same study contributed multiple effect sizes to a meta-analysis (e.g., more than one measure of interception), we divided the total number of study participants/the number of effect sizes the study contributed to the meta-analysis. In R (‘metafor’ package [36]), we conducted a weighted random effects meta-analyses to examine the pooled association between interoception and heavier body weight (two separate meta-analyses for studies in children only vs. studies in adults and mixed age). Forest plots and funnel plots were drawn using the ‘metaviz’ package in R. As we expected there to be too few prospective studies to meta-analyse, we planned to meta-analyse cross-sectional studies and describe the results of prospective studies narratively. We examined evidence for publication bias by examining asymmetry of the effect sizes after plotting and visually inspect funnel plots. Next, we used Egger’s test [37] and the Trim and Fill procedure [38] to statistically test for potential publication bias. We identified outliers and defined outliers as effects for which their 95% confidence interval was lower than the lower bound of the pooled effect confidence interval (i.e., extremely small effects) or for which the lower bound of the 95% confidence interval is higher than the upper bound of the pooled effect confidence interval (i.e., extremely large effects). To address influential cases, we planned to compute DFBETAS values [39] for each effect size. Influential cases were identified if DFBETAS values > 1 (indicative of a > 1 change in the standard deviation of the estimated co-efficient after removal of the effect from the meta-analysis [39]). To further increase sensitivity, we planned to conduct leave-one-out analyses by removing each effect size from the analyses and then refitting the model. See online supplementary materials for full information on the assessment of publication bias, outliers and influential cases. We also planned to conduct a series of analyses to address methodological quality and risk of bias of studies. We examined whether the results were similar to the primary analyses when the analyses were limited to studies that met key methodological criteria; (i) acceptable variability in participant BMI, (ii) accounted for participants with psychiatric disorders, (iii) did not use non-validated/standardised methods to measure interoception, all of (i)–(iii) and studies that used measured as opposed to self-report BMI (not pre-registered). We limited these analyses to studies that examined interoceptive accuracy in predominantly adult samples, as there were too few effects in children only samples and for other interoception facets when removing studies based on methodological criteria.

#### Secondary analyses

If we identified outliers in the primary analyses, we removed them for subsequent secondary analyses. We planned to conduct a series of meta-analyses in which we examined whether the relationship between interoception and BMI was moderated by interoceptive facet (i.e. accuracy vs. sensibility vs other) using sub-group analysis. We also examined whether the relationship between a facet of interoception (e.g., accuracy) and BMI differed based on how the facet was measured (e.g., heartbeat discrimination vs. heartbeat counting task). R code and data for analyses are available at https://osf.io/wfb96/.

## Results

### Search results

**Fig. 1:**
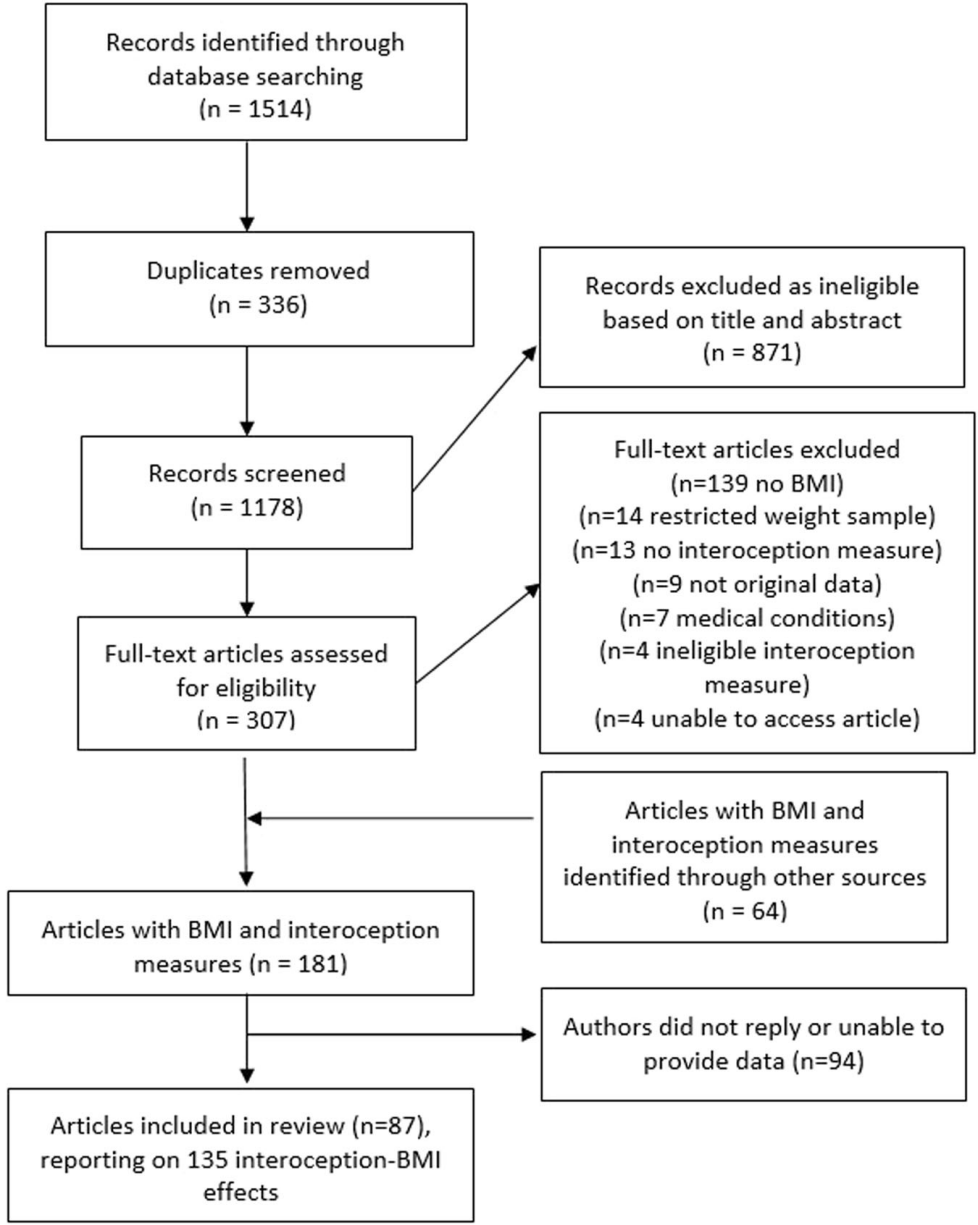
Flowchart of study search and selection process.

After removal of duplicates from electronic databases, the title and abstract of 1178 articles were screened (*n* = 871 removed during screening). We full-text screened 307 articles and of those 117 articles were eligible. We identified a further 64 eligible articles from grey literature, citation tracking and reference list searches. Of the 181 eligible articles, we were able to obtain effect size information on the relationship between interoception and BMI from 87 articles (statistical information was reported in 40 articles and after requests to authors we obtained the required statistical information from a further 47 articles). A total of 135 effect sizes were extracted from the 87 articles, as it was common for studies to include multiple measures of interoception. See Fig. 1 for the search process flowchart. See Online Supplementary Materials Document for bibliographic information of all included articles.

### Summary of included interoception–BMI effects

Participant age was reported for samples of the 129/135 interoception–BMI effects and mean age ranged from 8–67 years. The majority of included effects were from adult-only samples (*n* = 95), with *n* = 14 in children, *n* = 5 mixed samples and youngest participant age was not reported in *n* = 21 (although the mean values were indicative of adult samples). It was most common for effects to come from mixed-sex samples (*n* = 89), followed by females (*n* = 38) and male (*n* = 2) only samples (sex information missing in *n* = 6). The country in which a study was conducted was available for *n* = 126/135 and the most common origin was the USA (*n* = 29). Participant BMI summary information was available for *n* = 121/135. For a large proportion of the effects, no information was reported on how BMI was measured (*n* = 64) and similar numbers relied on self-reported weight and height to calculate BMI (*n* = 33) vs. researcher measurement (*n* = 37), *n* = 1 study used a combination. Participant sampling approach was available for *n* = 121/135 effects and it was most common for participants to be predominantly recruited from university campuses (*n* = 71). The largest study sample size was 1610 participants and the smallest 15 participants. Of all the effects included, *n* = 126/135 were correlational and *n* = 9/135 were group comparison (e.g., normal-weight participants vs. participants with obesity). The majority of included effects were cross-sectional (*n* = 133/135) and two studies examined the prospective relationship between a measure of interoception and weight change. Of the 135 BMI–interoception effects extracted, *n* = 77 measured interoception using objective task performance, *n* = 54 relied on self-report measures and for *n* = 4 a combination of the two was used (e.g., relationship between objective task performance and self-reported confidence in task performance). The most commonly examined facet of interoception was interoceptive accuracy (*n* = 89/135 effects) and *n* = 8 effects examined the relationship between interoceptive sensibility and BMI. The most common objective measure of interoceptive accuracy used was the heartbeat counting task (*n* = 52, including adaptations), followed by the heartbeat discrimination task (*n* = 12). For interoceptive sensibility, the most frequently used measure was Porges Body Perception Questionnaire (*n* = 4). Interoception awareness (e.g., heartbeat counting task accuracy vs. confidence ratings correspondence) was examined in *n* = 3 effects, one effect examined the relationship between interoception prediction error and BMI, and *n* = 3 effects examined gastric interoceptive sensitivity (water load task percentage satiation to total volume). The EDI/EDI-IA revised was available for *n* = 24 effects. See Online Supplementary Materials Document, Tables S1–S4 for individual study information.

#### Methodological quality and risk of bias

Coding for the six methodological quality and risk of bias indicators was consistent across authors (>90% agreement for each criteria) and inconsistencies were resolved through discussion. For 72/135 BMI–interoception effects, participants with a diagnosis of a psychiatric disorder were not included in the analyses, for 48/135 effects there was evidence of acceptable variability in BMI and for 20/135 effects participants with a BMI of <18.5 were excluded from analyses. A minority of effects (24/135) were drawn from studies with a very small sample size (defined as <20 participants per group in a group-comparison study and <30 participants for correlational designs). We identified potential measurement problems in 31/135 effects (e.g., non-standardised measures or task instructions/procedure) and the most effects (133/135) came from studies that were not pre-registered. See Online Supplementary Materials Document, Tables S5–S8 for individual study risk of bias ratings.

### Primary analyses

#### Child only samples

**Fig. 2:**
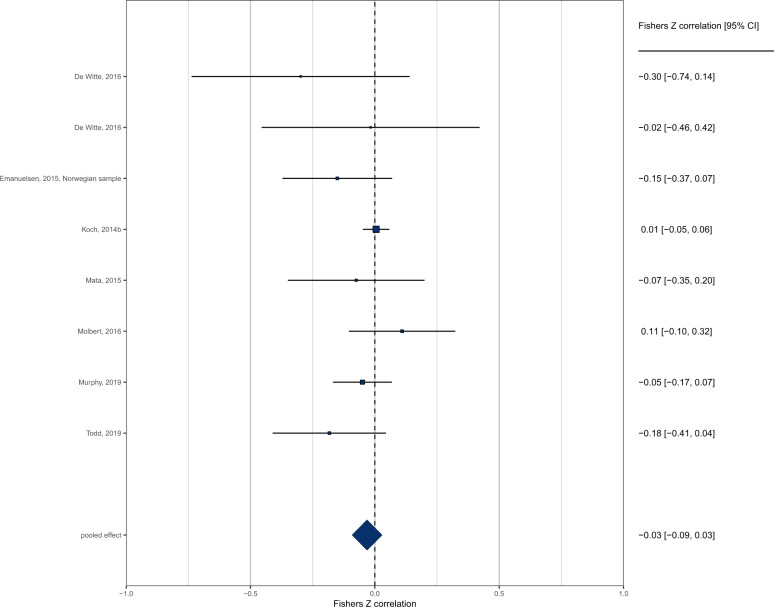
Forest plot of interoceptive accuracy and BMI meta-analysis in child-only samples.

**Fig. 3:**
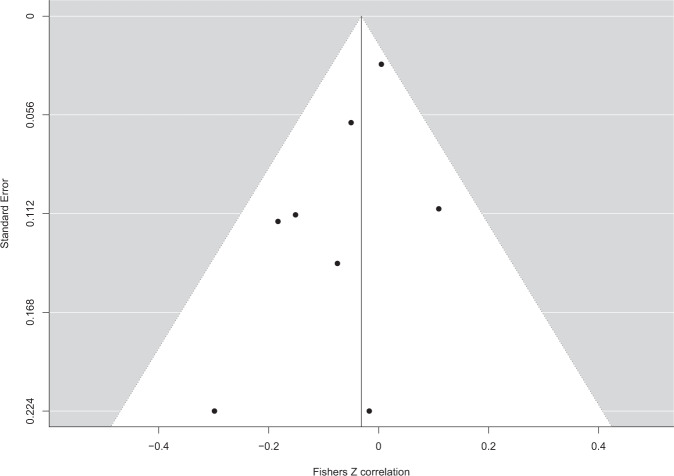
Funnel plot of interoceptive accuracy and BMI meta-analysis in child-only samples.

A total of *n* = 12 effects examined the cross-sectional relationship between interoception and BMI in children. Because only one study examined interoceptive sensibility, and three studies used ‘other’ measures (EDI), the meta-analysis was limited to interoceptive accuracy (8/12 effects). Pooled analysis of *n* = 8 effects (individual participants = 1974) demonstrated a non-significant negative association between interoception and BMI in children (pooled *r* = −0.031 (95% CI: −0.092 to 0.029), *z* = 1.01, *p* = .312, *I*^2^ = 13.8%) (Fig. 2). The funnel plot is shown in Fig. 3. Trim and fill imputed three studies (pooled *r* = −0.001 (95% CI: −0.044 to 0.041), *z* = 0.05, *p* = 0.959, *I*^2^ = 0.0%). Egger’s test was not significant (*z* = −1.58, *p* = .114). No influential cases or outliers were identified. Leave-one-out analyses demonstrated some fluctuation in the pooled correlations (range −0.066 to −0.011), but all models remained non-significant (*p*s > 0.101).

#### Adult samples

**Fig. 4:**
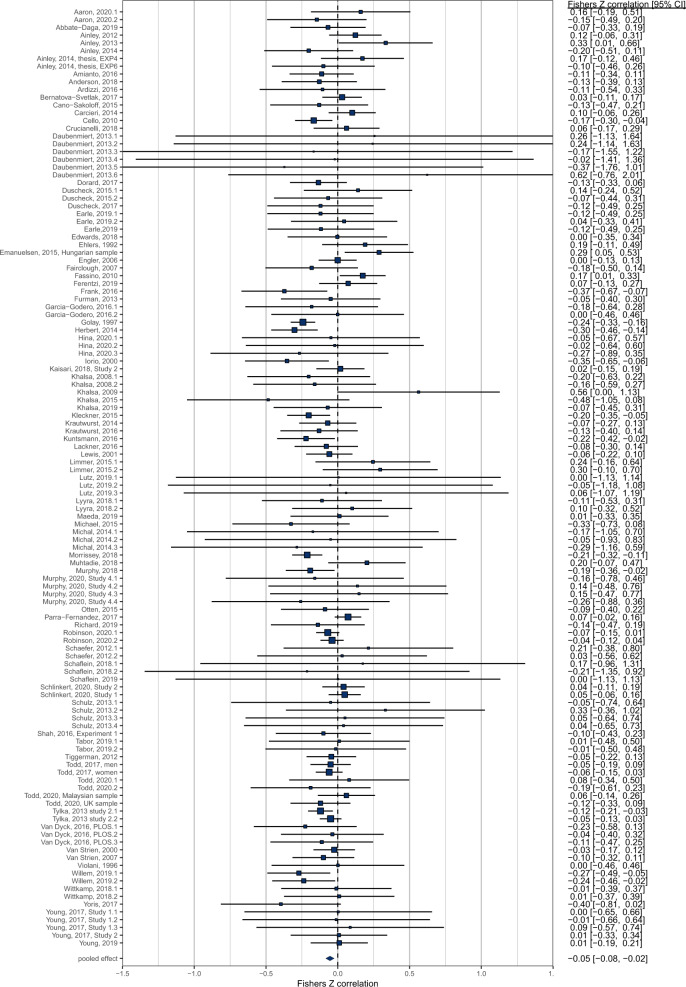
Forest plot of interoception and BMI meta-analysis in adult samples.

**Fig. 5:**
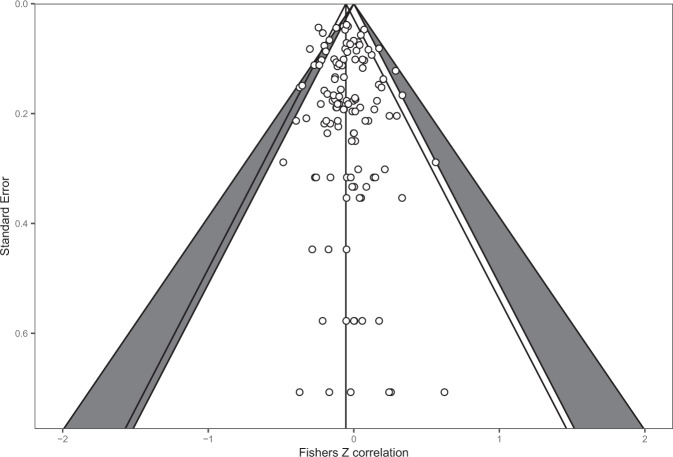
Funnel plot of interoception and BMI meta-analysis in adult samples.

There was a total of *n* = 121 effects examining the cross-sectional relationship between interoception and BMI in samples comprising of adults only or predominantly adult samples. Pooled analyses of *n* = 121 effects (individual participants = 10,425) demonstrated that there was a negative association between interoception and BMI (pooled *r* = −0.054 (95% CI: −0.084 to −0.025); *z* = 3.62, *p* < .001, *I*^2^ = 34.9%), whereby higher BMI was associated with deficits in interoception (Fig. 4). Visual inspection of the funnel plot (see Fig. 5) did not reveal substantial asymmetry. Trim and fill analyses imputed ten studies, which when included in the analyses slightly increased the association (pooled *r* = −0.068 (95% CI: −0.098 to −0.037), *z* = 4.38, *p* < 0.001, *I*^2^ = 38.1%). Egger’s test was not significant (z = 0.83, *p* = 0.406). No influential cases were identified. Eight effect sizes were identified as outliers with confidence intervals that did not overlap the pooled effect. Removing these outliers did not substantially influence the pooled correlation (pooled *r* = −0.056 (95% CI: −0.079 to −0.033) *z* = 4.80, *p* < 0.001, *I*^2^ = 2.9%), but did reduce the heterogeneity as expected. Leave-one-out analyses conducted on the data with outliers removed did not significantly influence the effect (pooled rs ranged from −0.060 to −0.052, *p* < 0.001).

### Secondary analyses

#### Moderation by interoception facet in adults

**Fig. 6:**
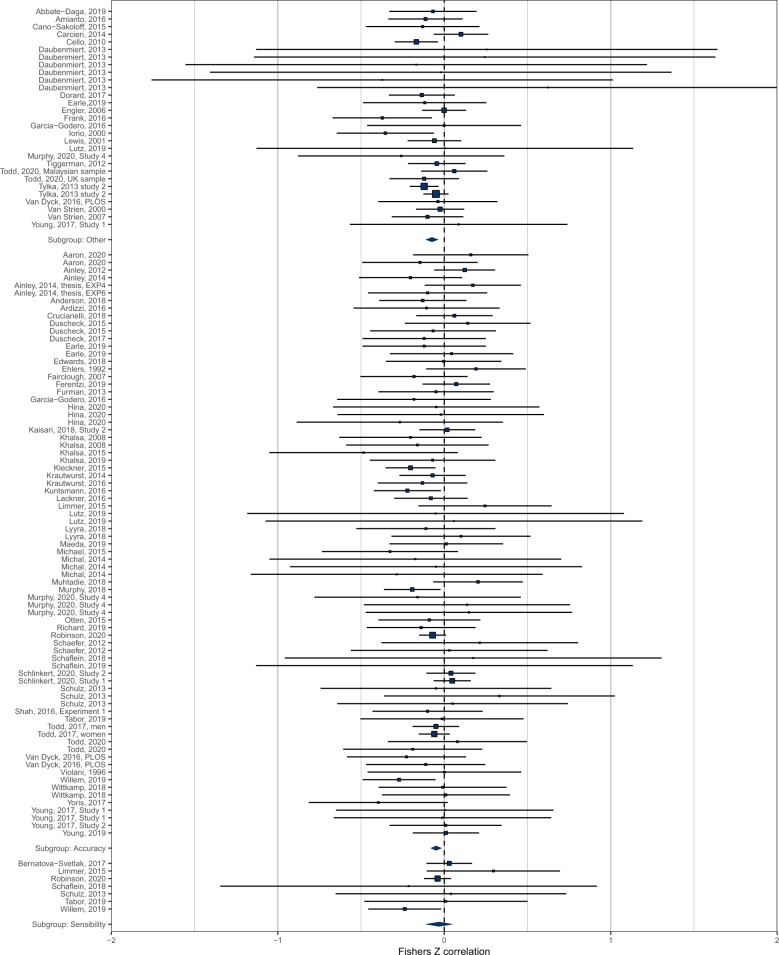
Forest plot of interoception sub-facet and BMI moderation analysis in adult samples.

Using the data from adults with outliers removed (*n* = 113), we examined interoception facet as a potential moderator of the association between interoception and BMI (Fig. 6). We included subgroups of accuracy (*n* = 77: pooled *r* = −0.049 (95% CI: −0.081 to −0.016), sensibility (*n* = 7: pooled *r* = −0.031 (95% CI: −0.112 to 0.051) and other (*n* = 29: pooled *r* = −0.074 (95% CI: −0.112 to −0.036). There was no significant moderation effect of interoception facet on the strength of the association between BMI and interoception (*X*^2^(2) = 1.47, *p* = 0.479).

#### Moderation by interoception measurement in adults

Using the data from adults with outliers removed, we examined whether the association between BMI and interoception was moderated by type of assessment of interoceptive accuracy (i.e., whether the relationship between interoceptive accuracy and BMI differs based on measure). We limited analyses to measures/assessments for which there were more than five effect sizes. There was no significant moderation effect (*X*^2^(2) = 1.79, *p* = 0.411). We did not conduct subgroup analyses by type of assessment for sensibility or other measures as there were too few effect sizes to compare. See Table 1 for the pooled associations between BMI and individual measures of interoception.

**Table 1:** Relationship between interoception and BMI for individual interoception measures.

	*n*	Pooled *r*	95% CIs		*Z*	*p* val	*I*^2^%
A. Accuracy							
Heartbeat count	42	−0.041	−0.088	0.005	1.73	0.082	4.5
Discrimination	12	−0.120	−0.219	−0.018	2.32	0.021	0.0
HCT confidence (sp)	8	−0.049	−0.208	0.113	0.60	0.551	0.0
IAS (sp)	2	−0.72	−0.151	0.009	1.75	0.080	0.0
BCQ (sp)	3	0.041	−0.061	0.143	0.80	0.424	0.0
MAIA noticing (sp)	5	−0.080	−0.152	−0.008	2.17	0.030	0.0
B. Sensibility							
Porges	5	0.009	−0.081	0.062	0.26	0.798	2.5
MAIA listening	2	−0.233	−0.424	−0.021	2.15	0.031	–
C. Other							
Confidence-accuracy	3	−0.105	−0.351	0.155	0.787	0.430	0
Water load	3	−0.028	−0.164	0.109	0.398	0.691	3.7
Respiratory	6	0.093	−0.440	0.578	0.322	0.747	0
EDI-IA	16	−0.079	−0.121	−0.038	3.73	0.001	9.1

*n* indicates number of effect sizes. Negative *r* value corresponds to higher BMI being associated with worse interoception (all interoception measures are standardised so that higher scores = better interoception).

*(sp)* self-perceived interoceptive accuracy. *Heartbeat count* heartbeat counting task, *Discrimination* heartbeat discrimination task, *EDI-IA* eating disorders inventory interoceptive awareness subscale, *HCT-confidence* heartbeat counting task confidence ratings, *IAS* interoceptive accuracy scale, *BCQ* body consciousness scale (private subscale), *Porges* Porges body perception scale (body awareness subscale), *MAIA* Multidimensional Assessment of Interoceptive Awareness Scale, *Confidence-Accuracy* correspondence between confidence and performance on heartbeat detection tasks, *Water load* water load task percentage satiation to total volume, *Respiratory* respiratory-based interoception perception tasks, *Prediction error* difference between interoceptive sensibility and interoceptive accuracy.

#### Methodological quality and risk of bias in cross-sectional associations between interoceptive accuracy and BMI

When examining only those studies in which participants with psychiatric disorders were excluded from analyses, the pooled correlation was (*n* = 53, *r* = −0.082 (95% CI: −0.127 to −0.037), *z* = 3.55, *p* < 0.001, *I*^2^ = 9.2%). Examining only studies with acceptable variability for BMI, the pooled correlation was (n = 30, r = −0.065 (95% CI: −0.123 to −0.008), *z* = 2.25, *p* = 0.025, *I*^2^ = 29.6%). Examining studies in which no potential measurement problems were identified, the pooled correlation was (*n* = 59, *r* = −0.055 (95% CI: −0.098 to −0.013), *z* = 2.55, *p* = 0.011, *I*^2^ = 24.8%). There were *n* = 11 effect sizes that were coded as having (1) sufficient variability in BMI; (2) did not include individuals with psychiatric disorders and; (3) had no potential measurement problems identified, for which the pooled correlation was (r = −0.122 (95% CI: −0.201 to −0.034), *z* = 2.72, *p* = 0.007, *I*^2^ = 30.0%). Examining results when measured BMI was available, the pooled correlation was (*n* = 15, *r* = −0.047 (95% CI: −0.133 to −0.040), *z* = 1.05, *p* = 0.293, *I*^2^ = 32.8%). Due to the methodological concerns over the heartbeat counting task, we examined the association between interoceptive accuracy and BMI using the heartbeat discrimination task (see Table 1 and online supplementary materials, figure S1) and results were consistent with the main analyses (*r* = −0.12).

### Unplanned exploratory analyses group-comparison studies

**Fig. 7:**
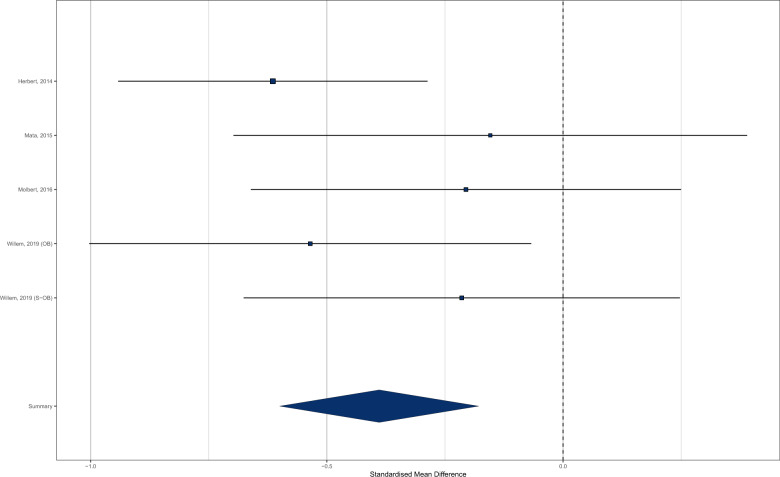
Forest plot of interoceptive accuracy meta-analysis for participants with normal weight vs. overweight/obesity.

For five effects (two studies of child-only samples and three studies of adults) there was a direct comparison between participants with normal weight and participants with overweight/obesity (BMI ≥ 25) for interoceptive accuracy. Consistent with the results of the main analyses, participants with overweight/obesity had worse interoceptive accuracy than did participants with normal weight (*N* normal weight = 221, *N* overweight/obese = 234; SMD = −0.389 (95% CI −0.600 to −0.178) *z* = 3.62, *p* < 0.001, *I*^2^ = 14.1%). See Fig. 7.

### Prospective associations between interoception and BMI

Koch et al. [29] examined the prospective relationship between interoception accuracy and BMI in a sample of German children (*N* = 1610) using the heartbeat counting task (higher values indicate greater interoception) and there was no significant association (*r* = −0.01, *p* > 0.05) of baseline interoceptive performance on follow-up BMI (measured one year later). Sehm et al. [40] examined the prospective association between interoception and BMI in a sample of German adolescents (*N* = 707) using the EDI. There was no significant relationship (*r* = −0.033, *p* > 0.39) between baseline interoception and follow-up BMI (measured 20 months later). See Online Supplementary Materials Document, Tables S4 and S8.

## Discussion

We conducted a systematic review and meta-analysis of the relationship between interoception and BMI. The majority of eligible studies were cross-sectional and meta-analyses of adult samples indicated that higher BMI is associated with deficits in interoception. In analyses limited to measures of interoceptive accuracy that came from studies with lower risk of bias, the negative relationship between BMI and interoception accuracy remained significant (*r* = −0.12) and was somewhat larger compared to the pooled analysis of all studies examining interoceptive accuracy (r = −0.05).

Although most studies in the adult sample meta-analysis examined interoceptive accuracy (measured with both objective tasks and self-report), we were able to include studies that examined other facets of interoception (e.g., interoceptive sensibility). We did not find convincing evidence that interoception facet type statistically moderated the association between interoception and BMI in adults, potentially indicating that higher BMI is associated with non-specific deficits in interoceptive processes. We also found no statistical evidence that the method used to assess interoceptive accuracy (e.g., heartbeat counting vs. discrimination tasks) significantly moderated the association between interceptive accuracy and BMI. However, a number of facets of interoception were not able to be included due to a lack of data (e.g., prediction error, awareness). Furthermore, the number of comparisons contributing to our moderation analyses was relatively small and therefore lack of moderation by interoception facet or measure type may be due to limited statistical power. As an illustrative example, the pooled effect size for the relationship between BMI and interoceptive accuracy measured using the heartbeat discrimination task (*n* = 12, *r* = −0.12) was small in statistical size but markedly larger than the pooled effect size for the relationship between BMI and interoceptive sensibility measured via the body perception questionnaire (*n* = 5, *r* = +0.009). It will therefore be important for future research to examine how different facets of interoception relate to BMI in order to assess whether higher BMI is associated with general or facet-specific deficits in interoception.

In child-only samples, there was only sufficient data to meta-analyse studies that measured interoceptive accuracy and although in line with the adult meta-analysis a weak negative association was observed, this was non-significant. This may reflect the small number of effects available, methodological difficulties associated with accurately measuring interoception in children or a lack of association between BMI and interoception prior to adulthood. Two studies of child and adolescent samples examined whether interoceptive accuracy was prospectively associated with BMI and there was no significant relationship in either study.

The most examined facet of interoception was interoceptive accuracy (both perceived and objectively measured) and the heartbeat counting task was the most frequently used task to measure interoceptive accuracy. A recent meta-analysis found a weak negative relationship between performance on the heartbeat counting task and BMI [41]. A number of methodological concerns been raised about the validity of the heartbeat counting task [28, 42], including that it does not differentiate between beliefs about resting heartrate and task measured heartbeat perception [43]. For this reason, it has been suggested that heartbeat discrimination tasks are more appropriate measures of interoceptive accuracy [28]. To address this concern, we examined the relationship between interoception and BMI in studies that used the heartbeat discrimination task and in line with our overall analysis, there was a significant negative relationship. A subset of studies used the EDI-IA self-report scale and there was evidence of deficits in interoception being associated with higher BMI in these studies. Although this scale has been widely used, it is not clear what total scores on this scale indicate. A number of items in the scale are more likely to measure eating disorder-specific symptomology (e.g. ‘I feel bloated after eating a normal meal’, ‘when I am upset, I worry that I will start eating’) and concerns have been raised over the extent to which the scale accurately measures interoception [44]. Few studies examined other facets of interoception, such as sensibility, awareness or prediction error. The extent to which these facets relate to BMI is therefore unclear. However, it is feasible that these underexplored facets of interoception may be of importance. For example, the meta-cognitive ‘awareness’ of whether one is accurately perceiving internal signals (e.g., ‘am I confident that this internal signal is hunger?’) may play an important role in shaping appetite regulation. Likewise, the extent to which there is concordance vs. discordance between interoceptive accuracy and sensibility (*prediction error*) might also underpin individual differences in appetite regulation, as eating in response to bodily signals may require alignment of both the ability to perceive signals and the tendency to listen to signals.

The most common modality of interoception examined was cardiac perception. A subset of studies examined interoception in relation to respiration [45] and gastric sensations [46], but the small number of effect sizes means that their relationship with BMI is unclear. Some work has suggested that interoceptive performance may transfer across modalities [46] and consistent with this deficits are observed across interoceptive modalities among individuals with eating disorders [16]. However, interoceptive detection accuracy across different sensory modalities is dissociable [47]. This consideration may be particularly important when considering interoception and obesity. Deficits in interoceptive processes that relate to perception of stomach distension, hunger and satiety signals may be more strongly associated with BMI than individual differences in modalities that are less directly relevant to appetite. Similarly, studies to date have tended to examine ‘static’ interoceptive processes, such as perceiving resting heartrate, as opposed to examining individual differences in the ability to identify dynamic changes in interoceptive signals. However, the ability to detect changes in interoceptive signals may be of particular importance to appetite regulation (e.g., changes in stomach distension as a result of eating).

The present meta-analyses were limited to cross-sectional studies and so temporal relationships between interoception and BMI cannot be inferred. It therefore remains unclear whether deficits in interoception are a cause or consequence of heavier body weight. Theoretically, it has been argued that deficits in interoception could result in internal appetite signals that encourage satiety being less strongly weighted into eating and food-related decision-making [16]. In individuals with a predisposition towards overeating, failure to integrate satiety signals into eating behaviour may in turn promote weight gain in the current food environment. Conversely, deficits in interoception are linked to emotion regulation problems and risky behaviour [48], which may increase the risk of maladaptive coping strategies (e.g., overeating) and in turn promote weight gain. However, higher BMI may also impact on interoception. For example, obesity is associated with a range of changes to underlying physiology [49], such as an attenuated or ‘blunted’ appetite response [50] and increased heartrate variability [51], and this may result in a range of interoceptive signals being ‘weaker’ or more difficult to perceive among individuals with obesity. Research addressing the temporal relationships between interoceptive processes and weight gain is needed to understand why deficits in interoception are associated with higher BMI.

The present research has several strengths. We were able to include a large number of studies and examine the relationship between interoception and BMI. A limitation of the review was that although we contacted authors of all eligible articles, we were unable to include effect sizes from several articles. There will likely be eligible studies that our search strategy was unable to identify due to the fact that interoception and BMI are often secondary measures and therefore less detectable through electronic searches. However, we found little evidence of publication bias and accounting for publication bias did not change the results. A limitation of interoception research is that there is a wide range of scales and measurement tools used to measure each individual facet of interoception and these measures may tap into different aspects of interoception. For example, studies used both the MAIA listening subscale and Porges body perception questionnaire and although this allowed us to characterise their independent and combined (‘sensibility’) associations with BMI, the correlation between the two measures is small [52]. It will therefore be important for future research to refine self-report measures of interoceptive sensibility. Likewise, we were able to characterise the independent and combined associations that measures of interoceptive accuracy (e.g., objective tasks such as the heartbeat counting and discrimination tasks, and self-reported perceived accuracy) have with BMI, but these measures also tend not to be strongly correlated and it is unclear whether measures of perceived accuracy are valid proxy measures of interoceptive accuracy. Given methodological issues surrounding the heartbeat counting task, we recommend that for the purpose of guiding future research the most appropriate effect size estimates reported here are for studies that have used the heartbeat discrimination task. Studies reported the raw correlation between interoception and BMI. Although in a set of analyses, we accounted for some potential confounding factors (e.g., diagnosis of mental health condition), it is plausible that there are other variables that are related to both interoception and BMI and may therefore act as confounders, such as subclinical depressive symptoms [53] or anxiety [47]. Future research would benefit from accounting for confounding variables when examining the relationships between interoception and BMI. Most studies included came from developed Western countries that have ‘obesogenic’ food environments and relatively high obesity prevalence (i.e., USA). Although BMI is a useful tool to characterise population levels of adiposity, interoception may be more strongly related to more direct measures of adiposity, such as body fat percentage or fat mass. Finally, it is important to note that the size of the statistical relationships between interoception and BMI in the main analyses tended to be statistically small, which is consistent with the relationship between obesity and a range of psychological variables [54]. However, for the studies that directly compared participants with normal weight vs. overweight/obesity on interoceptive accuracy across child and adult samples, the difference was somewhat larger than in the main analyses. This analysis was exploratory and based on a small number of studies, so caution in interpretation is required. In addition, the size of the relationship between interoception and BMI may be larger if other facets or types of interoceptive process (e.g., interoception relating to appetite/satiety signals) were examined.

## Conclusions

The results of this systematic review and meta-analysis indicate that higher BMI in adults is cross-sectionally associated with deficits in interoception, but the extent to which deficits are interoceptive facet-specific or non-specific is unclear. Further research is required to understand whether deficits in interoception contribute to and/or are a consequence of heavier body weight.

## Supplementary information

Online supplementary material
