# Supplementary material for: Interoception and obesity: a systematic review and meta-analysis of the relationship between interoception and BMI
Source: Int J Obes (Lond). 2021 Sep 3;45(12):2515–26. doi: 10.1038/s41366-021-00950-y (PMC8606313; doi:10.1038/s41366-021-00950-y)
Supplement: Supplementary file 1 — Online supplementary material [file 41366_2021_950_MOESM1_ESM.docx]

Online Supplementary Materials

Eligible and ineligible interoception measures

*Examples of ineligible measures of interoception*

Any interoceptive measures that do not directly measure one or more of the four interoceptive facets we are focusing on (accuracy, sensibility, awareness, prediction error) are ineligible. This includes measures that are likely to be confounded by other factors inherent to the nature of the task and will impact task performance. For example, the total amount of water consumed in a water load task (van Dyck et al., 2016) will be influenced by gastric capacity (which in turn is known to be associated with body weight) and therefore does not act as a direct measure of gastric interoception. Likewise, self-reported fullness/satiety measures after eating food and self-report measures including other processes (e.g. intuitive eating, emotion appraisal/regulation) are also ineligible, unless it is clear which facet of interoception is being directly measured. Brain imaging tasks are also ineligible as they do not provide data that can be used in meta-analysis to quantify the size of the statistical relationship (e.g. Pearson’s r) between facets of interoception and body weight. Other tasks, such as pain or temperature tolerance tasks (e.g. pain threshold test) are ineligible unless they have been modified to specifically test for objective interoception ability, as opposed to being confounded with ability to tolerate aversive stimuli. We will exclude studies that have used self-report global measures of interoception which comprise of items that tap into multiple facets of interoception (e.g. both accuracy and sensibility). For example, total score on the Body Awareness Questionnaire (Shields, Mallory, & Simon, 1989) is an ineligible measure because the total scale is multi-dimensional and measures inteorceptive accuracy as well as self-report information on sleep-wake cycles. However, the ‘Note response or change in body process’ subscale would qualify as a self-report measure of interoceptive accuracy. Likewise, total scores on the Multidimensional Assessment of Interoceptive Awareness (Mehling, Acree, Stewart, Silas, & Jones, 2018) would not be eligible, but subscale scores for Noticing (interoceptive accuracy) and Listening (interoceptive sensibility) would be eligible.

*Examples of common eligible measures of interoception*

Interoceptive accuracy (objective): Heartbeat discrimination task, Heartbeat counting task, inspiratory resistance detection task.

Interoceptive accuracy (self-report): Interoceptive Accuracy Scale (Murphy et al., 2019), Interoceptive Confusion Questionnaire (Brewer, Cook, & Bird, 2016), Confidence ratings from heartbeat counting task (without correspondence with actual performance), Interoceptive Awareness subscale of the Eating Disorders Inventory (EDI), including subsequent modifications, e.g. EDI2 (Garner, Olmstead, &Polivy, 1983), Noticing subscale of the Multidimensional Assessment of Interoceptive Awareness (MAIA and subsequent versions).

Interoceptive sensibility (self-report): Porge’s Body Perception Questionnaire, including shortened versions, e.g. BPQ-Short Form (Cabrera et al., 2018), Body listening subscale of the Multidimensional Assessment of Interoceptive Awareness (MAIA and subsequent versions).

Interoceptive awareness (objective and self-report): Confidence-accuracy correspondence when completing the heartbeat counting task.

Interoceptive prediction error (objective and self-report): Discrepancy between objective performance on an interoceptive accuracy task and self-reported awareness, i.e. difference between Z scored values for each task, as in (Garfinkel et al., 2016) and (Young et al., 2017).

Search terms

(interocept* OR heartbeat detection OR heartbeat counting OR heartbeat discrimination OR heartbeat awareness) AND (health OR BMI OR body mass index OR body weight OR Overweight OR obesity). Search terms used for PubMed, SCOPUS and PsycINFO on the 10/02/20. For PUBMED we also applied the ‘human’ participants filter when searching.

Risk of bias

1) *Diagnosis of psychiatric disorder/eating disorders*

There is evidence of differences in interoception in people with major psychiatric disorders (e.g. depression, anxiety, eating disorders) and psychiatric disorders are also associated with body weight. We will therefore record whether studies have accounted for psychiatric disorders (i.e. through eligibility criteria or removal prior to analyses).

*2) Variability in body weight*

To be able to examine the association between interoception and heavier body weight there needs to be a sufficient variability in body weight in a sample of participants. In studies that have used a group comparison design (recruitment of a group of participants with normal weight vs. a group of participants with overweight/obesity) this issue does not typically apply. However, in correlational studies (interoception correlated with continuous body weight) a limited range of body weight and/or very few overweight/obese participants is problematic. We will therefore record information on the range and variability of body weight included in such studies (e.g. SD of sample body weight, range of sample body weight, proportion of participants with OV/OB) in order to evaluate whether each study is likely to suffer from a lack of variability in body weight. We will assess whether (or not) each study has insufficient variability in body weight based on reported data:

*Lack of information:* If a study reports no information about variability of BMI it will be coded as having unclear variability.

*Minimum and maximum BMI values*: if a study reports that the maximum BMI value was below the obese weight range (≥30) it will be coded as having low variability.

*Proportion of participants with heavier body weight*: if a correlational study reports that only a small minority of participants were of heavier body weight (i.e. less than 25% of participants were considered to be heavier than normal weight; BMI ≥25) it will be coded as having low variability.

*Standard deviation (SD) of BMI*: if a correlational study reports data that indicate a relatively small SD (<4 BMI points) it will be coded as having low variability.

*Reasonable variability*: For a study to be coded as having reasonable variability it is required to have reported data for at least one of the above three criteria and for that data to indicate reasonable variability (i.e. SD ≥4 BMI points). If a study has reported data on more than one criteria all reported data is required to indicate reasonable variability.

*3) Accounting for U shaped body weight deficits in interoception*

Very low body weight is thought to be associated with deficits in interception ^1^, which results in a theoretical U shaped deficit curve of interoception and body weight (deficits at lower body weight and higher body weight). Therefore, simply correlating interoception with body weight may produce misleading findings if a subset of the study population are underweight. However, studies that have reported the correlation between interoception and BMI may have no exclusion criteria for participants who are underweight (BMI < 18.5) when recruiting participants or not exclude such participants from analyses. We will therefore record whether (or not) studies have accounted for lower body weight, measured as a BMI < 18.5 (i.e. through eligibility criteria or removal in analyses).

*4)Whether the study had a very small sample size*

Very small sample sizes increase the likelihood of sporadic findings. Consistent with ^2 3^, for group comparison studies we operationalise a very small sample size as less than 20 participants per group in a group comparison study (total n=40) and less than 30 participants for correlational designs (total n=30). In both cases sporadic findings are a concern because: a study would be powered to detect a large effect sizes only and the small number of observations would increase likelihood of influential cases and/or random error having a disproportionate influence in analyses.

*5)Evidence of potential interoceptive measurement problems*

For self-report and objective measures of interoception we will assess whether there is any evidence of potential measurement problems. For example, in self-report measures whether an adapted (e.g. shortened, translated) but un-validated version of a scale has been used instead of the validated scale. For example, in objective measures whether only a single trial (or the number of trials was unspecified) on the heartbeat counting task or heartbeat discrimination was administered would be classed as a potential measurement problem, as would a modified/non-standardised version of the task that may affect performance. In addition, if there is insufficient information provided about the measurement and/or procedure used to assess interoception, we will code this as evidence of potential improper administration of interoceptive measure.

*6)Whether the study method and analysis plan was pre-registered.*

We will code whether or not the study refers to a pre-registered (prior to data collection) method and analysis plan.

Full information on outliers, influential cases and publication bias

*Outlier removal and influential case analyses*

We examined evidence for outliers in the primary analysis. We characterise outliers as any effect sizes: which the upper bound of the 95% confidence interval is lower than the lower bound of the pooled effect confidence interval (i.e., extremely small effects) or for which the lower bound of the 95% confidence interval is higher than the upper bound of the pooled effect confidence interval (i.e., extremely large effects). If any outliers were identified we planned to report the results of meta-analyses with the outliers included and removed. To address influential cases, we also computed DFBETAS values for each effect size. Influential cases will be identified if DFBETAS values > 1 (indicative of a >1 change in the standard deviation of the estimated co-efficient after removal of the study ^4^. To increase sensitivity, we also conducted leave-one-out analyses by removing each study (k) from the analyses, and refitting the model. If the removal of k substantially influences the model (statistical significance of the effect changes from p < .05 to p> .05 (or p >.05 to p < .05), this was classed as an influential case.

*Publication bias*

We examined publication bias in primary analyses by examining asymmetry of the effect sizes. We first plotted and visually inspect funnel plots for potential publication bias. Next, we planned to conduct an Egger’s test of asymmetry and Trim and Fill procedure. Egger’s test ^5^ (examines the regression coefficient between the Standard Normal Deviate (effect size/standard error) and the estimate’s precision (1/standard error). If the intercept is significantly different from 0 at p >.10 this is indicative of bias. Trim and Fill ^6^ removes less precise studies which might case any asymmetry (‘trim’), re-estimates the overall effect size, and then replaces removed studies and missing counterparts (‘fill’) required for symmetry. We planned to report i) the number of missing (‘filled’) studies, and ii) the estimate of the effect size following their inclusion.

Online Supplementary Materials 2: Study details

Table S1. Summary data for cross-sectional studies examining interoceptive accuracy (and EDI-IA) and BMI

| Author and year | Age  (M and SD) | Gender | Country conducted in | BMI (M and SD) | Measure of interoception | Number of pps in analysis | Effect size^^[[1]](#footnote-2)^^ |
| --- | --- | --- | --- | --- | --- | --- | --- |
| Aaron, 2020^7^ | 19.70 (0.95) | 50 F, 26 M | US | Not reported | Heartbeat counting task (objective) | 71 | r=0.158 |
| Aaron, 2020^7^ | 19.70 (0.95) | 50 F, 26 M | US | Not reported | Heartbeat counting task confidence judgements (Self-report) | 69 | r=-0.145 |
| Abbate-Daga, 2019^8^ | 25.08 (3.23) | 59 F | Italy | 20.64 (2.01) | EDI-II IA (self-report) | 59 | r=-0.68 |
| Ainley, 2012^9^ | 28.7 (13.5) | 74 F, 55 M | UK | 23.1 (4.3) | Heartbeat counting task (objective) | 118 | r=0.122 |
| Ainley, 2013^10^ | 21.04 (1.33) | 39 F | UK | 19.69 (2.20) | Heartbeat counting task (objective) | 39 | r=0.322 |
| Ainley, 2014^11^ | 19.6 (4.9) | 34 F, 9 M | UK | 22.09 (3.45) | Heartbeat counting task (objective) | 43 | r=-0.20 |
| Ainley, 2015, exp 4, thesis^12^ | 21.0 (3.1) | 36 F, 10 M | UK | 22.02 (4.30) | Heartbeat counting task (objective) | 49 | r=0.170 |
| Ainley, 2015, exp 6, thesis^12^ | 20.3 (3.0) | 24 F, 4 M | UK | 23.00 (4.46) | Heartbeat counting task (objective) | 33 | r=-0.100 |
| Aminato, 2016^13^ | 23.28 (0.63) | 80 F | Italy | 21.95 (2.46) | EDI-II IA (self-report) | 80 | r=-0.112 |
| Anderson, 2018^14^ | 19.09 (1.54) | 59 F | US | 23.78 (4.11) | Heartbeat counting task (objective) | 59 | r=-0.128 |
| Ardizzi, 2016^15^ | 31.91 (9.18) | 3 F, 20 M | Italy | 23.27 (2.54) | Heartbeat counting task (objective) | 23 | r=-0.106 |
| Cano Sakoloff, 2015^16^ | 19.7 (2.4) | 36 F | US | 21.8 (2.4) | EDI-II IA (self-report) | 36 | r=-0.129 |
| Carcieri, 2014^17^ | 19.19 (1.93) | 146 F | US | 23.99 (4.08) | EDI IA (self-report) | 146 | r=0.10 |
| Cella, 2010^18^ | 28.17 | 61 F, 170 M | Italy | 24.93 | EDI-II IA (self-report) | 231 | r=-0.166 |
| Crucianelli, 2018^19^ | 22.07 (2.75) | 76 F | UK | Not reported | Heartbeat counting task (objective) | 76 | r=0.061 |
| Daubenmier, 2013^20^ | 41.9 (11) | 7 F, 11 M | US | 23.7 (3.4) | Respiratory load detection task (objective) | 18 | r=0.250 |
| Daubenmier, 2013^20^ | 41.9 (11) | 7 F, 11 M | US | 23.7 (3.4) | Respiratory load discrimination task (objective) | 15 | r=0.238 |
| Daubenmier, 2013^20^ | 41.9 (11) | 7 F, 11 M | US | 23.7 (3.4) | Respiratory tracking task (objective) | 18 | r=-0.167 |
| Daubenmier, 2013^20^ | 35.4 (11.4) | 8 F, 8 M | US | 23.2 (3.4) | Respiratory load detection task (objective) | 16 | r=-0.213 |
| Daubenmier, 2013^20^ | 35.4 (11.4) | 8 F, 8 M | US | 23.2 (3.4) | Respiratory load discrimination task (objective) | 16 | r=-0.356 |
| Daubenmier, 2013^20^ | 35.4 (11.4) | 8 F, 8 M | US | 23.2 (3.4) | Respiratory tracking task (objective) | 16 | r=0.553 |
| De Witte, 2016^21^ | 13 (2.13) | 27 F, 19 M | Belgium | Not reported | Heartbeat counting task (objective) | 46 | r=-0.29 |
| De Witte, 2016^21^ | 13 (2.13) | 27 F, 19 M | Belgium | Not reported | Heartbeat counting task confidence judgement (self-report) | 46 | r=-0.017 |
| Dorard, 2017^22^ | 35.28 (9.76) | 65 F, 36 M | France | 24.12 (4.04) | EDI-II IA (self-report) | 101 | r=0.134 |
| Duschek, 2015^23^ | 24.45 (3.4) | 40 F, 20 M | Spain | 21.7 (2.5) | Heartbeat counting task (objective) | 60 | r=-0.067 |
| Duschek, 2015^23^ | 24.45 (3.4) | 40 F, 20 M | Spain | 21.7 (2.5) | Body consciousness questionnaire, private body consciousness (self-report) | 60 | r=0.14 |
| Duschek, 2017^24^ | 47.13 (9.38) | 31 F | Spain | 25.41 (4.41) | Heartbeat counting task (objective) | 31 | r=-0.12 |
| Edwards, 2018^25^ | 21.52 (5.12) | 14 F, 21 M | UK | 24 (5.42) | Heartbeat counting task (objective) | 35 | r=-0.003 |
| Ehlers, 1992, study 2^26^ | 31.7 (7.3) | 34 F, 12 M | Germany | 22.3 (2.5) | Heartbeat counting task (objective) | 46 | r=-0.188 |
| Emanuelsen, 2015^27^ | 21.54 (2.40) | 49 F, 21 M | Hungry | 21.41 (2.44) | Heartbeat counting task (objective) | 70 | r=0.28 |
| Emanuelsen, 2015^27^ | 17.3 (1.59) | 53 F, 29 M | Norway | 21.68 (2.99) | Heartbeat counting task (objective) | 82 | r=-0.15 |
| Engler, 2006^28^ | 19.4 (3.6) | 223 F | US | 22.61 (3.33) | EDI IA (self-report) | 223 | r=-0.001 |
| Erle, 2019^29^ | 24.60 (4.59) | 82 F, 18 M | Germany | 23.01 (3.90) | Heartbeat counting task (objective) | 96 | r=-0.12 |
| Erle, 2019^29^ | 24.60 (4.59) | 82 F, 18 M | Germany | 23.01 (3.90) | Heartbeat counting task confidence judgements (self-report) | 96 | r=-0.117 |
| Fairclough, 2007^30^ | 25.55 (5.6) | 20 F, 20 M | Not reported | Not reported | Heartbeat discrimination task (objective) | 40 | r=-0.18 |
| Fassino, 2010^31^ | 24.36 (3.6) | 154 F | Italy | 20.04 (1.6) | EDI-II IA (self-report) | 154 | r=0.172 |
| Ferenzti, 2019^32^ | 21.9 (3.6) | 66 F, 76 M | Hungary | 22.61 (2.83) | Heartbeat counting task (objective) | 97 | r=0.072 |
| Frank, 2016^33^ | 27.03 (7.55) | 46 F | US | Healthy weight 21.5 (1.4).  Obese 34.7 (4.6) | EDI IA (self-report) | 46 | r=-0.356 |
| Furman, 2013^34^ | 36.0 (12.5) | 36 F | US | 23.8 (3.3) | Heartbeat counting task (objective) | 35 | r=-0.0497 |
| Garcia-Cordero, 2016^35^ | 66.90 (7.45) | 26 F, 16 M | Not reported | 26.30 (3.64) | Heartbeat counting task (objective) | 42 | r=-0.18 |
| Golay, 1997^36^ | 44.65 (13.12) | 533 F | Switzerland | Control 22.75.  Obese 33.35 | EDI IA (self-report) | 533 | r=-0.23 |
| Herbert, 2014^37^ | 24.82 | 110 F, 40 M | Germany | Overweight/Obese= 27.2 (SD=2), Normal weight range 18.5- 25 | Heartbeat counting task (objective) | 150 | r=-0.293 |
| Hina, 2020^38^ | 23.75 (5.87) | 21 F, 19 M | UK | 24.565 (4.21) | Heartbeat discrimination task (objective) | 40 | r=-0.021 |
| Hina, 2020^38^ | 23.75 (5.87) | 21 F, 19 M | UK | 24.565 (4.21) | Heartbeat counting task (objective) | 40 | r=-0.261 |
| Hina, 2020^38^ | 23.75 (5.87) | 21 F, 19 M | UK | 24.565 (4.21) | MAIA, noticing subscale (self-report) | 40 | r=-0.048 |
| Iorio, 2020^39^ | 36.6 (6.54) | 48 F | Italy | Normal weight 22.23 (1.40).  Overweight 25.43 (0.92) | EDI IA (self-report) | 48 | r=-0.34 |
| Kaisari, 2018^40^ | 19.3 (1.19) | 114 F, 28 M | UK | 21.4 (3.57) | Heartbeat counting task (objective) | 140 | r=0.018 |
| Khalsa, 2008^41^ | 50.64 (9.38) | 31 F, 16 M | US | 23.82 (4.64) | Heartbeat discrimination task (objective) | 47 | r=-0.20 |
| Khalsa, 2008^41^ | 50.64 (9.38) | 31 F, 16 M | US | 23.82 (4.64) | Heartbeat discrimination task, confidence judgements (self-report) | 47 | r=-0.16 |
| Khalsa, 2009^42^ | 37.0 (12.8) | 5 F, 10 M | US | 25.2 (3.4) | Average cross correlation between mean centred dial ratings and instantaneous heart rate changes in response to bolus (iso) infusion (objective) | 15 | r=0.51 |
| Khalsa, 2015^43^ | 22.3 (4.5) | 15 F | US | 22.7 (3.7) | Average cross correlation between mean centred dial ratings and instantaneous heart rate changes in response to bolus (iso) infusion (objective) | 15 | r=-0.45 |
| Khalsa, 2019^44^ | 44.35 (13.4) | 10 F, 20 M | US | 25.0 (4.3) | Average cross correlation between mean centred dial ratings and instantaneous heart rate changes in response to bolus (iso) infusion (objective) | 30 | r=-0.07 |
| Kleckner, 2015^45^ | 24.09 (7.05) | 97 F, 77 M | US | 24.22 (5.45) | Heartbeat discrimination task (objective) | 174 | r=-0.20 |
| Koch, 2014 b^46^ | 8.39 (0.94) | 693 F, 657 M | Germany | 16.99 (2.63) | Heartbeat counting task (objective) | 1350 | r=0.005 |
| Krautwurst, 2014^47^ | 23.7 (3.4) | 79 F, 21 M | Germany | 21.6 (2.8) | Heartbeat counting task (objective) | 100 | r=-0.07 |
| Krautwurst, 2016^47^ | Not reported | Not reported | Germany | Not reported | Heartbeat counting task (objective) | 56 | r=-0.13 |
| Kunstmann, 2016^48^ | 18.50 (0.77) | 101 F | US | 22.71 (3.34) | Heartbeat counting task (objective) | 98 | r=-0.218 |
| Lackner, 2016^49^ | 19.63 (2.04) | 75 F, 26 M | US | 26.70 (6.16) | Heartbeat counting task (objective) | 82 | r=-0.08 |
| Lewis, 2001^50^ | Not reported | 151 F | US | 138 Normal weight.  13 Obese | EDI IA (self-report) | 151 | r=-0.059 |
| Limbrunner, 2004^51^ | Not reported | 262 F | US | 23.2 | EDI IA (self-report) | 262 | r=0.197 |
| Limmer, 2015^52^ | 41 (14) | 38 F, 15M | Germany | 25.1 (7.5) | Product-moment correlation between 4 self-report measures of arousal and physio arousal data (objective) | 53 | r=0.240 |
| Lutz, 2019^53^ | 24.60 (3.94) | 19 F | Luxembourg | 22.33 (3.19) | Heartbeat counting task (objective) | 19 | r=-0.052 |
| Lutz, 2019^53^ | 24.60 (3.94) | 19 F | Luxembourg | 22.33 (3.19) | Heartbeat counting task confidence judgement (self-report) | 19 | r=0.058 |
| Lutz, 2019^53^ | 24.60 (3.94) | 19 F | Luxembourg | 22.33 (3.19) | EDI-II IA (self-report) | 19 | r=-0.004 |
| Lyyra, 2018^54^ | 24.46 (3.89) | 27 F, 23 M | Finland | 22.28 (2.14) | Heartbeat discrimination task (objective) | 50 | r=-0.11 |
| Lyyra, 2018^54^ | 24.46 (3.89) | 27 F, 23 M | Finland | 22.28 (2.14) | Heartbeat discrimination task confidence ratings (self-report) | 50 | r=0.10 |
| Maeda, 2019^55^ | 21.7 (1.7) | 20 F, 16 M | Japan | 21.09 (2.32) | Heartbeat counting task (objective) | 36 | r=0.012 |
| Mata, 2015^56^ | 15.37 | 33 F, 21 M | Spain | Overweight=29.40 (3.00).  Normal weight 21.17 (2.24) | Heartbeat counting task (objective) | 54 | r=-0.075 |
| Michael, 2015^57^ | 21.9 (2.8) | 19 F, 7 M | France | 21.5 (2.7) | Heartbeat counting task (objective) | 26 | r=-0.315 |
| Michal, 2014^58^ | 26.4 (1.6) | 12 F, 14 M | Germany | 21.4 (2.4) | Heartbeat counting task (objective) | 26 | r=-0.172 |
| Michal, 2014^58^ | 26.4 (1.6) | 12 F, 14 M | Germany | 21.4 (2.4) | Heartbeat discrimination task (objective) | 24 | r=-0.050 |
| Michal, 2014^58^ | 26.4 (1.6) | 12 F, 14 M | Germany | 21.4 (2.4) | KEKS (self-report) | 26 | r=-0.278 |
| Molbert, 2016^59^ | 12.87 (1.58) | 44 F, 43 M | Germany | BMI SDS  Obese 2.51 (SD= 0.6).  Normal weight -0.2 (SD=0.6) | Heartbeat counting task (objective) | 87 | r=0.22 |
| Morrissey, 2018^60^ | 16.14 (2.93) | 353 F | US | 22.24 (4.46) | EDI IA (self-report) | 353 | r=-0.21 |
| Muhtadie, 2018^61^ | 22.7 (5.8) | 34 F, 22 M | US | 23.6 (4.4) | Modified body awareness questionnaire (self-report) | 56 | r=0.20 |
| Murphy, 2018^62^ | 55.10 (19.50) | 87 F, 49 M | UK | Not reported | Heartbeat counting task (objective) | 136 | r=-0.194 |
| Murphy, 2019^63^ | 8.47 (0.18) | 159 F, 120 M | UK | 15.53 (2.56) | Heartbeat counting task (objective) | 278 | r=-0.050 |
| Murphy, 2020, study 4^64^ | Not reported | Not reported | Not reported, MTURK | 26.50 (7.38) | Heartbeat counting task (objective) | 53 | r=0.136 |
| Murphy, 2020, study 4^64^ | Not reported | Not reported | Not reported, MTURK | 26.50 (7.38) | Heartbeat counting task confidence judgements (self-report) | 53 | r=0.147 |
| Murphy, 2020, study 4^64^ | Not reported | Not reported | Not reported, MTURK | 26.50 (7.38) | Interoceptive accuracy scale (self-report) | 53 | r=-0.159 |
| Otten, 2015^65^ | 39.0 (8.0) | 24 F, 20 M | Germany | 21.75 (2.5) | Heartbeat counting task (objective) | 44 | r=-0.089 |
| Parra-Fernandez, 2018^66^ | 21.74 (4.73) | 295 F, 159 M | Spain | 22.21 | EDI-II IA (self-report) | 454 | r=-0.071 |
| Richard, 2019^67^ | 22.0 (3.55) | 39 F | Austria | 21.8 (1.76) | Heartbeat counting task (objective) | 39 | r=-0.138 |
| Robinson, 2020^68^ | 37.2 (12.6) | 576 F, 605 M | UK | 26.3 (4.9) | Interoceptive accuracy scale (self-report) | 1181 | r=-0.07 |
| Schaefer, 2012^69^ | 41.74 (12.52) | 16 F, 11 M | Germany | 24.55 (3.82) | Heartbeat counting task (objective) | 27 | r=0.21 |
| Schaefer, 2012^69^ | 41.74 (12.52) | 16 F, 11 M | Germany | 24.55 (3.82) | Heartbeat discrimination task (objective) | 27 | r=0.03 |
| Schaflein, 2018^70^ | 41.1 (10.0) | 17 F, 1 M | Germany | 24.7 (2.9) | Heartbeat counting task (objective) | 18 | r=0.172 |
| Schaflein, 2018^70^ | 41.1 (10.0) | 17 F, 1 M | Germany | 24.7 (2.9) | MAIA, noticing subscale (self-report) | 18 | r=0.001 |
| Schlinkert, 2020, study 1^71^ | 35 (11) | 222 F, 90 M | US | Not reported | Body Consciousness Questionaire, private body subscale (self-report) | 312 | r=0.048 |
| Schlinkert, 2020 study 2^71^ | 20 | 148 F, 32 M | The Netherlands | 21.6 (2.8) | Heartbeat counting task, non-auditory condition (objective) | 180 | r=0.040 |
| Schulz, 2013^72^ | 22.95 (2.55) | 29 F, 13 M | Germany | 22.05 (2.9) | Heartbeat counting task (objective) | 42 | r=-0.05 |
| Schulz, 2013^72^ | 22.95 (2.55) | 29 F, 13 M | Germany | 22.05 (2.9) | Heartbeat discrimination task, auditory (objective) | 42 | r= 0.32 |
| Schulz, 2013^72^ | 22.95 (2.55) | 29 F, 13 M | Germany | 22.05 (2.9) | Heartbeat discrimination task, visual (objective) | 42 | r=0.05 |
| Sehm, 2015^73^ | 14.44 (1.60) | 308 F, 371 M | Germany | BMI SDS 0.15 (1.00) | EDI-C IA (self-report) | 672 | r=0.038 |
| Shah, 2016^74^ | 29.1 (12.7) | 20 F, 18 M | UK | Not reported | Heartbeat counting task (objective) | 38 | r=-0.10 |
| Tabor, 2019^75^ | 23 (4) | 19 F, 19 M | UK | 22.07 (2.43) | Heartbeat counting task (objective) | 38 | r=-0.014 |
| Tiggeman, 2012^76^ | 20.4 (2.87) | 146 F | Australia | 23.61 (4.93) | EDI IA (self-report) | 132 | r=-0.045 |
| Todd adolescents, 2019^77^ | 14.11 (1.01) | 140 F, 125 M | UK | Not reported | MAIA, noticing subscale (self-report) | 153 | r=-0.181 |
| Todd, 2019, adults^78^ | 37.76 (11.97) | 199 M | UK | 27.76 (6.46) | MAIA, noticing subscale (self-report) | 199 | r=-0.05 |
| Todd, 2019, adults^78^ | 39.46 (11.57) | 446 F | UK | 26.10 (4.97) | MAIA, noticing subscale (self-report) | 446 | r=-0.06 |
| Todd, 2020^79^ | 26.08 (6.73) | 28 F, 21 M, 1 other | UK | 5.38 (4.91) | Heartbeat counting task, confidence judgements (self-report) | 50 | r=-0.187 |
| Todd, 2020^79^ | 26.08 (6.73) | 28 F, 21 M, 1 other | UK | 24.24 (4.91) | Heartbeat counting task (objective) | 50 | r=0.079 |
| Tylka, 2013, study 2^80^ | 20.45 (5.06) | 520 M | US | 25.38 (5.48) | EDI-II IA (self-report) | 520 | r=0.12 |
| Tylka, 2013, study 2^80^ | 20.45 (5.06) | 680 F | US | 24.02 (5.68) | EDI-II IA (self-report) | 680 | r=0.05 |
| Van Dyck, 2016^81^ | 22.86 (3.41) | 99 F | Luxembourg | 22.73 (3.60) | Heartbeat counting task (objective) | 95 | r=-0.223 |
| Van Dyck, 2016^81^ | 22.86 (3.41) | 99 F | Luxembourg | 22.73 (3.60) | Body Consciousness Questionaire, private body subscale (self-report) | 99 | r=-0.111 |
| Van Strien, 2000^82^ | 21.2 (3.2) | 200 F | The Netherlands | 21.1 (2.16) | EDI-R IA (self-report) | 187 | r=-0.025 |
| Van Strein, 2005^83^ | 15.6 (1.5) | 436 F | The Netherlands | 20.06 (2.6) | EDI-R IA (self-report) | 694 | r=-0.119 |
| Van Strein, 2007^84^ | 21.1 (1.88) | 86 F | The Netherlands | 23.0 (3.45) | EDI-II IA (self-report) | 86 | r=-0.10 |
| Violani, 1996^85^ | Not reported | 21 F | Italy | Not reported | Heartbeat discrimination task (objective) | 21 | r=0.001 |
| Willem, 2019^86^ | 41.83 | 126 F, 39 M | France | Normal weight 21.9  Moderately obese 35.49  Severely obese 45.54 | MAIA, noticing subscale (self-report) | 165 | r=-0.265 |
| Wittkamp, 2018^87^ | 23.4 (3.5) | 40 F, 19 M | Luxembourg | 22.4 (3.0) | Heartbeat counting task (objective) | 59 | r=-0.01 |
| Wittkamp, 2018^87^ | 23.4 (3.5) | 40 F, 19 M | Luxembourg | 22.4 (3.0) | Heartbeat discrimination task, visual (objective) | 59 | r=0.01 |
| Yoris, 2018^88^ | 65.92 (7.51) | 17 F, 9 M | Argentina | 25.39 (3.27) | Heartbeat counting task (objective) | 25 | r=-0.377 |
| Young, 2017, study 1^89^ | 21.87 (1.76) | 36 F | UK | 23.7 (4.28) | Heartbeat counting task (objective) | 36 | r=0.003 |
| Young, 2017, study 1^89^ | 21.87 (1.76) | 36 F | UK | 23.7 (4.28) | Heartbeat counting task, confidence judgements (self-report) | 36 | r=-0.011 |
| Young, 2017, study 2^89^ | 20.56 (1.80) | 37 F | UK | 22.5 (4.33) | Heartbeat discrimination task (objective) | 37 | r=0.008 |
| Young, 2019^90^ | 20.7 (1.9) | 100 F | UK | 24.1 (4.3) | Heartbeat counting task (objective) | 100 | r=0.010 |

Table S2. Summary data for studies examining interoceptive sensibility and BMI with cross-sectional designs^[[2]](#footnote-3)^

| Author and year | Age  (M and SD) | Gender | Country | BMI  (M and SD) | Measure of interoception | Number of pps in analysis | Effect size |
| --- | --- | --- | --- | --- | --- | --- | --- |
| Bernatova-Svetlak, 2017^91^ | 22.32 (2.42) | 207 F | Czech Republic | 22.3 (3.5) | Porges body perception questionnaire, awareness subscale (self-report) | 207 | r=0.03 |
| Limmer, 2015^52^ | 41 (14) | 38 F, 15 M | Germany | 25.1 (7.5) | Porges body perception questionnaire, awareness subscale (self-report) | 53 | r=0.287 |
| Robinson, 2020^68^ | 37.2 (12.6) | 576 F, 605 M | UK | 26.3 (4.9) | Porges body perception questionnaire, awareness subscale-Short form (self-report) | 1181 | r= -0.04 |
| Schaflein, 2018^70^ | 41.1 (10.0) | 17 F, 1 M | Germany | 24.7 (2.9) | MAIA, body listening subscale (self-report) | 18 | r=-0.211 |
| Schulz, 2013^72^ | 22.95 (2.55) | 29 F, 13 M | Germany | 22.05 (2.9) | Porgesbody perception questionnaire, awareness subscale (self-report) | 42 | r=0.04 |
| Tabor, 2019^75^ | 23 (4) | 19 F, 19 M | UK | 22.07 (2.43) | Porges Body perception questionnaire, awareness subscale (self-report) | 38 | r=0.10 |
| Todd adolescents, 2019^77^ | 14.11 (1.01) | 140 F, 125 M | UK | Not reported | MAIA, body listening subscale (self-report) | 153 | r=-0.048 |
| Willem, 2019^86^ | 41.83 | 126 F, 39 M | France | Normal weight 21.9  Moderately obese 35.49  Severely obese 45.54 | MAIA, body listening subscale (self-report) | 165 | r=-0.233 |

Table S3. Summary data for studies examining other facets of interoception (awareness, prediction error, gastric sensitivity) and BMI with cross-sectional designs^[[3]](#footnote-4)^

| Author and year | Age  (M and SD) | Gender | Country | BMI  (M and SD) | Measure of interoception (objective vs. self-report vs. mixed) | Number of pps in analysis | Effect size |
| --- | --- | --- | --- | --- | --- | --- | --- |
| Erle, 2019^29^ | 24.60 (4.59) | 82 F, 18 M | Germany | 23.01 (3.90) | Heartbeat counting task accuracy- confidence correspondence (mixed) | 86 | r=-0.117 |
| Garcia-Cordero, 2016^35^ | 66.90 (7.45) | 26 F, 16 M | Not reported | 26.30 (3.64) | Modified heartbeat counting task accuracy-confidence correspondence (mixed) | 42 | r=0.001 |
| Murphy, 2020  study 4^64^ | Not reported | Not reported | Not reported | 26.50 (7.38) | Heartbeat counting task accuracy-confidence correspondence (mixed) | 53 | r=-0.253 |
| Todd, 2020^79^ | 25.47 (8.40) | 54 F, 37 M | UK | 25.64 (5.74) | Waterload percentage satiation to total volume (objective) | 91 | r=-0.12 |
| Todd, 2020^79^ | 22.63 (3.25) | 50 F, 50 M | Malaysia | 23.56 (5.18) | Waterload percentage satiation to total volume (objective) | 100 | r=0.06 |
| Van Dyck, 2016^81^ | 22.86 (3.41) | 99 F | Luxembourg | 22.73 (3.60) | Waterload percentage satiation to total volume (objective) | 99 | r=-0.038 |
| Young, 2017, study 1^89^ | 21.87 (1.76) | 36 F | UK | 23.7 (4.28) | Prediction error (mixed) | 36 | r=0.086 |

Table S4. Summary data for studies examining interoceptionand subsequent BMI (prospective studies)^[[4]](#footnote-5)^

| Author and year | Age  (M and SD) | Gender | Country | BMI  (M and SD) | Measure of interoception | Number of pps in analysis | Effect size |
| --- | --- | --- | --- | --- | --- | --- | --- |
| Koch, 2014 a^92^ | 9.13 (0.93) | 836 F, 774 M | Germany | BMI SDS 0.19 (1.00) | Heartbeat counting task (objective) | 1610 | r=-0.01 |
| Sehm, 2018^93^ | 15.85 (1.50) | Not reported | Germany | BMI SDS 18 (1.06) | EDI-C IA (self-report) | 707 | r=-0.033 |

Table S5. Further methodological information for studies examining interoceptive accuracy (or EDI-IA) and BMI (cross-sectional design)^[[5]](#footnote-6)^

| Author and year | Child or adult sample | BMI measure | Sample | Measure of interoception | Number of trials | Analysis adjusted | Diagnosis PC/ED excluded | BMI variance | UW pps removed | Very small sample size | Measure problems |
| --- | --- | --- | --- | --- | --- | --- | --- | --- | --- | --- | --- |
| Aaron, 2020^7^ | Adult | Self-report | University students | Heartbeat counting task (objective) | 3 | N | Y | U | U | N | N |
| Aaron, 2020^7^ | Adult | Self-report | University students | Heartbeat counting task, confidence judgement (self-report) | 3 | N | Y | U | U | N | N |
| Abbate-Daga, 2019^8^ | Adult | Measured | University sample | EDI-II IA (self-report) | N/A | N | Y | N | U | N | N |
| Ainley, 2012^9^ | Mixed | Not reported | Visitors of science museum | Heartbeat counting task (objective) | 6 | N | U | Y | U | N | Y |
| Ainley, 2013^10^ | Adult | Not reported | University students | Heartbeat counting task (objective) | 3 | N | U | N | U | N | N |
| Ainley, 2014^11^ | Adult | Not reported | University students | Heartbeat counting task (objective) | 3 | N | U | U | U | N | N |
| Ainley, 2015 exp 4^12^ | Adult | Self-report | University students | Heartbeat counting task (objective) | 3 | N | U | Y | U | N | N |
| Ainley, 2015, exp 6^12^ | Adult | Self-report | University students | Heartbeat counting task (objective) | 3 | N | U | Y | U | N | N |
| Amianto, 2016^13^ | Adult | Not reported | University sample | EDI-II IA (self-report) | N/A | N | U | N | U | N | N |
| Anderson, 2018^14^ | Adult | Measured | Community | Heartbeat counting task (objective) | 4 | N | Y | Y | N | N | N |
| Ardizzi, 2016^15^ | Not reported | Not reported | Not reported | Heartbeat counting task (objective) | 4 | N | Y | N | U | Y | N |
| Cano Sakoloff, 2015^16^ | Mixed | Measured | Non-athletes | EDI-II IA (self-report) | N/A | N | U | N | U | N | N |
| Carcieri, 2014^17^ | Adult | Measured | University students | EDI IA (self-report) | N/A | N | U | Y | N | N | N |
| Cella, 2010^18^ | Adult | Measured | Homosexuals (110) and Heterosxuals(121) during events and demonstrations | EDI-II IA (self-report) | N/A | N | U | U | U | N | N |
| Crucianelli, 2018^19^ | Adult | Not reported | University sample | Heartbeat counting task (objective) | 3 | N | Y | U | U | N | N |
| Daubenmier, 2013^20^ | Not reported | Not reported | Meditation centres | Respiratory load detection task (objective) | 35 | N | U | N | U | Y | N |
| Daubenmier, 2013^20^ | Not reported | Not reported | Meditation centres | Respiratory load discrimination task (objective) | 36 | N | U | N | U | Y | N |
| Daubenmier, 2013^20^ | Not reported | Not reported | Meditation centres | Respiratory tracking task (objective) | Continuous measurement | N | U | N | U | Y | N |
| Daubenmier, 2013^20^ | Not reported | Not reported | Community | Respiratory load detection task (objective) | 35 | N | U | N | U | Y | N |
| Daubenmier, 2013^20^ | Not reported | Not reported | Community | Respiratory load discrimination task (objective) | 36 | N | U | N | U | Y | N |
| Daubenmier, 2013^20^ | Not reported | Not reported | Community | Respiratory tracking task (objective) | Continuous measurement | N | U | N | U | Y | N |
| De Witte, 2016^21^ | Child | Measured | Children form schools and youth groups | Heartbeat counting task (objective) | 6 | N | Y | U | U | N | Y |
| De Witte, 2016^21^ | Child | Measured | Children form schools and youth groups | Heartbeat counting task, confidence judgements (self-report) | N/A | N | Y | U | U | N | Y |
| Dorard, 2017^22^ | Adult | Self-report | Online | EDI-II IA (self-report) | N/A | N | U | Y | N | N | N |
| Duschek, 2015^23^ | Adult | Self-report | Not reported | Heartbeat counting task (objective) | 3 | N | Y | N | U | N | N |
| Duschek, 2015^23^ | Adult | Self-report | Not reported | Body consciousness questionnaire, private body consciousness (self-report) | N/A | N | Y | N | U | N | N |
| Duschek, 2017^24^ | Adult | Not reported | Local women’s associations | Heartbeat counting task (objective) | 3 | N | Y | Y | U | N | Y |
| Edwards, 2018^25^ | Adult | Not reported | University students | Heartbeat counting task (objective) | 4 | Y | U | Y | U | N | Y |
| Ehlers, 1992, study 2^26^ | Not reported | Not reported | Local community | Heartbeat counting task (objective) | 6 | N | Y | N | U | N | N |
| Emanuelsen, 2015^27^ | Adult | Measured | Hungarian University students | Heartbeat counting task (objective) | 3 | N | U | N | U | N | N |
| Emanuelsen, 2015^27^ | Child | Measured | Norwegian high school students | Heartbeat counting task (objective) | 3 | N | U | N | U | N | N |
| Engler, 2006^28^ | Adult | Measured | University students | EDI IA (self-report) | N/A | N | U | N | U | N | N |
| Erle, 2019^29^ | Adult | Not reported | Community, mainly students | Heartbeat counting task (objective) | 4 | N | U | N | Y | N | N |
| Erle, 2019^29^ | Adult | Not reported | Community, mainly students | Heartbeat counting task, confidence judgements (self-report) | N/A | N | U | N | Y | N | N |
| Fairclough, 2007^30^ | Not reported | Self-report | Not reported | Heartbeat discrimination task (objective) | 48 | N | Y | U | U | N | N |
| Fassino, 2010^31^ | Mixed | Not reported | University sample | EDI-II IA (self-report) | N/A | N | Y | N | U | N | N |
| Ferentzi, 2018^32^ | Adult | Not reported | University students | Heartbeat counting task (objective) | 3 | N | U | N | N | N | N |
| Frank, 2016^33^ | Adult | Not reported | Not reported | EDI IA (self-report) | N/A | N | U | Y | Y | Y | N |
| Furman, 2013^34^ | Adult | Measured | Local community | Heartbeat counting task (objective) | 3 | N | Y | N | U | N | N |
| Garcia-Cordero, 2016^35^ | Not reported | Not reported | Not reported | Heartbeat counting task (objective) | Unclear(continuous trial) | N | Y | N | U | N | Y |
| Golay, 1997^36^ | Adult | Measured | Outpatient samples | EDI IA (self-report) | N/A | N | Y | Y | Y | N | N |
| Herbert, 2014^37^ | Adult | Not reported | University sample | Heartbeat counting task (objective) | 4 | Y | Y | Y | Y | N | N |
| Hina, 2020^38^ | Adult | Not reported | University sample and community | Heartbeat discrimination task (objective) | 16 | N | Y | Y | N | N | N |
| Hina, 2020^38^ | Adult | Not reported | University sample and community | Heartbeat counting task (objective) | 4 | N | Y | Y | N | N | N |
| Hina, 2020^38^ | Adult | Not reported | University sample and community | MAIA, noticing subscale (self-report) | N/A | N | Y | Y | N | N | N |
| Iorio, 2000^39^ | Adult | Measured | Mothers of primary school children | EDI IA (self-report) | N/A | N | U | Y | Y | N | Y |
| Kaisari, 2018^40^ | Adult | Measured | University students | Heartbeat counting task (objective) | 2 | N | U | N | N | N | N |
| Khalsa, 2008^41^ | Adult | Not reported | Experienced meditators | Heartbeat discrimination task (objective) | 92 | N | Y | Y | U | N | Y |
| Khalsa, 2008^41^ | Adult | Not reported | Experienced meditators | Heartbeat discrimination task (objective) | N/A | N | Y | Y | U | N | Y |
| Khalsa, 2009^42^ | Adult | Measured | Not reported | Average cross correlation between mean centred dial ratings and instantaneous heart rate changes in response to bolus (iso) infusion (objective) | 6 | N | Y | N | Y | Y | Y |
| Khalsa, 2015^43^ | Not reported | Not reported | Not reported | Average cross correlation between mean centred dial ratings and instantaneous heart rate changes in response to bolus (iso) infusion (objective) | 7 | N | Y | N | U | Y | Y |
| Khalsa, 2019^44^ | Adult | Not reported | Meditators and non-meditators | Average cross correlation between mean centred dial ratings and instantaneous heart rate changes in response to bolus (iso) infusion (0bjective) | 6 | N | Y | Y | U | N | Y |
| Kleckner, 2015^45^ | Adult | Mixed | University sample | Heartbeat discrimination task (objective) | 100 | N | Y | Y | N | N | N |
| Koch, 2014 b^46^ | Child | Measured | Primary school students | Heartbeat counting task (objective) | 3 | N | U | U | U | N | Y |
| Krautwurst, 2014^47^ | Adult | Not reported | University students | Heartbeat counting task (objective) | 3 | N | Y | N | N | N | N |
| Krautwurst, 2016^47^ | Not reported | Not reported | Community sample | Heartbeat counting task (objective) | 3 | N | Y | U | U | N | N |
| Kunstmann, 2016^48^ | Adult | Measured | University students | Heartbeat counting task (objective) | 3 | N | U | N | U | N | N |
| Lackner, 2016^49^ | Adult | Measured | University students | Heartbeat counting task (objective) | 6 | N | U | Y | U | N | N |
| Lewis, 2001^50^ | Adult | Self-report | Community | EDI IA (self-report) | N/A | N | U | Y | Y | Y | N |
| Limbrunner, 2004^51^ | Child | Self-report | High school students | EDI IA (self-report) | N/A | N | U | U | U | N | N |
| Limmer, 2015^52^ | Not reported | Self-report | Community sample | Product-moment correlation between 4 self-report measures of arousal and physio arousal data (objective) | Continuous measurement | N | N | Y | U | N | Y |
| Lutz, 2019^53^ | Adult | Not reported | University students | Heartbeat counting task (objective) | 4 | N | Y | N | U | Y | N |
| Lutz, 2019^53^ | Adult | Not reported | University students | Heartbeat counting task confidence judgements (self-report) | 4 | N | Y | N | U | Y | N |
| Lutz, 2019^53^ | Adult | Not reported | University students | EDI-II IA (self-report) | N/A | N | Y | N | U | Y | N |
| Lyyra, 2018^54^ | Adult | Self-report | University students, experience of HDT | Heartbeat discrimination task (objective) | 96 | N | U | N | U | N | Y |
| Lyyra, 2018^54^ | Adult | Self-report | University students, experience of HDT | Heartbeat discrimination task, confidence judgements (self-report) | N/A | N | U | N | U | N | Y |
| Maeda, 2019^55^ | Adult | Not reported | University sample | Heartbeat counting task (objective) | 6 | N | Y | N | U | N | N |
| Mata, 2015^56^ | Child | Not reported | Hospitals and local schools | Heartbeat counting task (objective) | 6 | N | Y | Y | Y | N | N |
| Michael, 2015^57^ | Adult | Self-reported | University students | Heartbeat counting task (objective) | 3 | N | Y | N | N | Y | N |
| Michal, 2014^58^ | Not reported | Not reported | University sample | Heartbeat counting task (objective) | 7 | N | U | N | U | Y | N |
| Michal, 2014^58^ | Not reported | Not reported | University sample | Heartbeat discrimination task (objective) | 20 | N | U | N | U | Y | Y |
| Michal, 2014^58^ | Not reported | Not reported | University sample | KEKS (self-report) | N/A | N | U | N | U | Y | Y |
| Molbert, 2016^59^ | Child | Measured | Community and children in weight loss program | Heartbeat counting task (objective) | 4 | N | Y | Y | Y | N | N |
| Morrissey, 2018^60^ | Mixed | Measured | Local community | EDI IA (self-report) | N/A | N | U | N | N | N | N |
| Muhtadie, 2018^61^ | Adult | Not reported | University psychology students | Adapted Body Awareness Questionnaire (self-report) | N/A | N | U | Y | U | N | Y |
| Murphy, 2018^62^ | Adult | Measured | Not reported | Heartbeat counting task (objective) | 4 | N | Y | U | U | N | N |
| Murphy, 2019^63^ | Child | Not reported | Previous study cohort | Heartbeat counting task (objective) | 3 | N | Y | N | N | N | N |
| Murphy, 2020, study 4^64^ | Adult | Self-report | MTURK | Heartbeat counting task (objective) | 4 | N | U | Y | N | N | N |
| Murphy, 2020, study 4^64^ | Adult | Self-report | MTURK | Heartbeat counting task, confidence judgement (self-report) | N/A | N | U | Y | N | N | N |
| Murphy, 2020, study 4^64^ | Adult | Self-report | MTURK | Interoceptive accuracy scale (self-report) | N/A | N | U | Y | N | N | N |
| Otten, 2018^65^ | Adult | Not reported | University sample and meditators | Heartbeat counting task (objective) | 4 | Y | U | N | N | N | N |
| Parra-Fernandez, 2018^66^ | Adult | Self-report | University students | EDI-II IA (self-report) | N/A | N | U | N | N | N | N |
| Richard, 2019^67^ | Mixed | Not reported | University sample | Heartbeat counting task (objective) | 6 | N | Y | N | Y | N | N |
| Robinson, 2020^68^ | Adult | Self-report | Online panel | Interoceptive accuracy scale (self-report) | N/A | N | Y | Y | Y | N | N |
| Schaefer, 2012^69^ | Not reported | Not reported | Not reported | Heartbeat counting task (objective) | 3 | N | Y | N | U | Y | N |
| Schaefer, 2012^69^ | Not reported | Not reported | Not reported | Heartbeat discrimination task (objective) | 60 | N | Y | N | U | Y | N |
| Schaflein, 2018^70^ | Adult | Not reported | University sample | Heartbeat counting task (objective) | 3 | N | Y | N | Y | Y | N |
| Schaflein, 2018^70^ | Adult | Not reported | University sample | MAIA, noticing subscale (self-report) | N/A | N | Y | N | Y | Y | N |
| Schlinkert, 2020 study 1^71^ | Adult | Self-report | MTURK | Body Consciousness Questionnaire, Private Body subscale (self-report) | N/A | N | U | U | U | N | N |
| Schlinkert, 2020, study 2^71^ | Adult | Self-report | University students | Heartbeat counting task, visual condition (objective) | 3 | N | Y | N | U | N | Y |
| Schulz, 2013^72^ | Adult | Not reported | University students | Heartbeat counting task (objective) | 3 | N | Y | N | U | N | N |
| Schulz, 2013^72^ | Adult | Not reported | University students | Heartbeat discrimination task, auditory (objective) | 20 | N | Y | N | U | N | N |
| Schulz, 2013^72^ | Adult | Not reported | University students | Heartbeat discrimination task, visual (objective) | 20 | N | Y | N | U | N | N |
| Sehm, 2015^73^ | Child | Measured | Not reported, adolescents | EDI-C IA (self-report) | N/A | N | Y | U | U | N | N |
| Shah, 2016^74^ | Adult | Measured | Participant database | Heartbeat counting task (objective) | 4 | N | Y | U | U | N | N |
| Tabor, 2019^75^ | Adult | Not reported | University sample | Heartbeat counting task (objective) | 3 | N | Y | N | N | N | N |
| Tiggeman, 2012^76^ | Adult | Not reported | University students | EDI IA (self-report) | N/A | N | U | Y | N | N | N |
| Todd, adolescents, 2019^77^ | Child | Self-report | Secondary school students | MAIA, noticing subscale (self-report) | N/A | N | U | U | U | N | N |
| Todd, adults 2019^78^ | Adult | Self-report | Online, Male | MAIA, noticing subscale (self-report) | N/A | N | U | Y | N | N | N |
| Todd, adults 2019^78^ | Adult | Self-report | Online, Female | MAIA, noticing subscale (self-report) | N/A | N | U | Y | N | N | N |
| Todd, 2020^79^ | Adult | Not reported | University sample | Heartbeat counting task, confidence ratings (self-report) | N/A | N | Y | Y | N | N | Y |
| Todd, 2020^79^ | Adult | Not reported | University sample | Heartbeat counting task (objective) | 5 | N | Y | Y | N | N | Y |
| Tylka, 2013^80^ | Adult | Self-report | University students | EDI-II IA (self-report) | N/A | N | U | Y | N | N | N |
| Tylka, 2013^80^ | Adult | Self-report | University students | EDI-II IA (self-report) | N/A | N | U | Y | N | N | N |
| Van Dyck, 2016^81^ | Adult | Measured | University sample | Heartbeat counting task (objective) | 4 | N | Y | N | N | N | N |
| Van Dyck, 2016^81^ | Adult | Measured | University sample | Body consciousness questionnaire, PBC subscale (self-report) | N/A | N | Y | N | N | N | N |
| Van Strein, 2000^82^ | Adult | Not reported | University sample | EDI-R IA (self-report) | N/A | N | U | N | U | N | N |
| Van Strien, 2005^83^ | Child | Self-report | School students | EDI-R IA (self-report) | N/A | N | U | N | U | N | N |
| Van Strien, 2007^84^ | Adult | Measured | University sample | EDI-II IA (self-report) | N/A | N | U | N | U | N | Y |
| Violani, 1996^85^ | Adult | Not reported | University students | Heartbeat discrimination task (objective) | 40 | N | U | U | U | Y | Y |
| Willem, 2019^86^ | Adult | Self-report | University sample, and diabetology and bariatric centres | MAIA, noticing subscale (self-report) | N/A | N | U | Y | U | N | N |
| Wittkamp, 2018^87^ | Adult | Not reported | University and high school students | Heartbeat counting task (objective) | 3 | N | Y | N | N | N | Y |
| Wittkamp, 2018^87^ | Adult | Not reported | University and high school students | Heartbeat discrimination task, visual (objective) | 40 | N | Y | N | N | N | Y |
| Yoris, 2018^88^ | Not reported | Not reported | Not reported | Heartbeat counting task (objective) | 2 continuous measurements | N | Y | N | U | Y | Y |
| Young, 2017, study 1^89^ | Adult | Measured | Not reported | Heartbeat counting task (objective) | 5 | N | Y | Y | Y | N | N |
| Young, 2017, study 1^89^ | Adult | Measured | Not reported | Heartbeat counting task, confidence judgements (self-report) | N/A | N | Y | Y | Y | N | N |
| Young, 2017, study 2^93^ | Adult | Measured | Not reported | Heartbeat discrimination task (objective) | 48 | N | Y | Y | N | Y | N |
| Young, 2019^90^ | Adult | Measured | Not reported | Heartbeat counting task (objective) | 5 | N | Y | Y | N | N | N |

Table S6. Further methodological information for studies examining interoceptive sensibility and BMI (cross-sectional design)^[[6]](#footnote-7)^

| Author and year | Child or adult sample | BMI measure | Sample | Measure of interoception | Number of trials | Analysis adjusted | Diagnosis PC/ED excluded | BMI variance | UW pps removed | Very small sample size | Measure problems |
| --- | --- | --- | --- | --- | --- | --- | --- | --- | --- | --- | --- |
| Bernatova-Svetlak, 2017 ^91^ | Adult | Not reported | University students and graduates | Porgesbody perception questionnaire, awareness subscale (self-report) | N/A | N | U | N | U | N | N |
| Limmer, 2015^52^ | Not reported | Self-report | Community sample | Porgesbody perception questionnaire, awareness subscale (self-report) | N/A | N | N | Y | U | N | N |
| Robinson, 2020^68^ | Adult | Self-report | Online panel | Porges body perception questionnaire, awareness subscale-Short form (self-report) | N/A | N | Y | Y | Y | N | N |
| Schaflein, 2018^70^ | Adult | Not reported | University sample | MAIA, body listening subscale (self-report) | N/A | N | Y | N | Y | Y | N |
| Schulz, 2013^72^ | Adult | Not reported | University students | Porgesbody perception questionnaire, awareness subscale (self-report) | N/A | N | Y | N | U | N | N |
| Tabor, 2019^75^ | Adult | Not reported | University community | Porgesbody perception questionnaire, awareness subscale (self-report) | N/A | N | Y | N | N | N | N |
| Todd, adolescents, 2019^77^ | Child | Self-report | Secondary school students | MAIA, body listening subscale (self-report) | N/A | N | U | U | U | N | N |
| Willem, 2019^86^ | Adult | Self-report | University sample, and diabetology and bariatric centres | MAIA, body listening subscale (self-report) | N/A | N | U | Y | U | N | N |

Table S7. Further methodological information for studies examining interoceptive awareness, prediction error and gastric sensitivity and BMI (cross-sectional design)^[[7]](#footnote-8)^

| Author and year | Child or adult sample | BMI measure | Sample | Measure of interoception | Number of trials | Analysis adjusted | Diagnosis PC/ED excluded | BMI variance | UW pps removed | Very small sample size | Measure problems |
| --- | --- | --- | --- | --- | --- | --- | --- | --- | --- | --- | --- |
| Erle, 2019^29^ | Adult | Not reported | Community | Heartbeat counting task accuracy-confidence correspondence (mixed) | 4 | N | U | N | Y | N | N |
| Garcia-Codero, 2016^35^ | Not reported | Not reported | Not reported | Modified heartbeat counting task accuracy-confidence correspondence (mixed) | Unclear (continuous trial) | N | Y | N | U | N | Y |
| Murphy, 2020, study 4^64^ | Adult | Self-report | MTURK | Heartbeat counting task accuracy-confidence correspondence (mixed) | 4 | N | U | Y | N | N | N |
| Todd, 2020^79^ | Adult | Measured | University sample (UK) | Waterload percentage satiation to total volume (objective) | N/A | N | Y | Y | N | N | N |
| Todd, 2020^79^ | Adult | Measured | University sample (Malaysia) | Waterload percentage satiation to total volume (objective) | N/A | N | Y | Y | N | N | N |
| Van Dyck, 2016^81^ | Adult | Measured | University sample | Waterload percentage satiation to total volume (objective) | 1 | N | Y | N | N | N | N |
| Young, 2017, study 1^89^ | Adult | Measured | Female only | Heartbeat counting task accuracy-confidence correspondence (mixed) | N/A | N | Y | Y | Y | N | N |

Table S8. Further methodological information for studies examining interoceptionand subsequent BMI (prospective study designs)^[[8]](#footnote-9)^

| Author and year | Child or adult sample | BMI measure | Sample | Measure of interoception | Number of trials | Analysis adjusted | Diagnosis PC/ED excluded | BMI variance | UW pps removed | Very small sample size | Measure problems |
| --- | --- | --- | --- | --- | --- | --- | --- | --- | --- | --- | --- |
| Koch, 2014 a^92^ | Child | Measured | Primary school students | Heartbeat counting task (objective) | 3 | N | U | N | U | N | Y |
| Sehm, 2018^93^ | Child | Measured | Not reported | EDI-C IA (objective) | N/A | N | U | U | U | N | N |

Figure S1. Association between BMI and heartbeat discrimination task performance


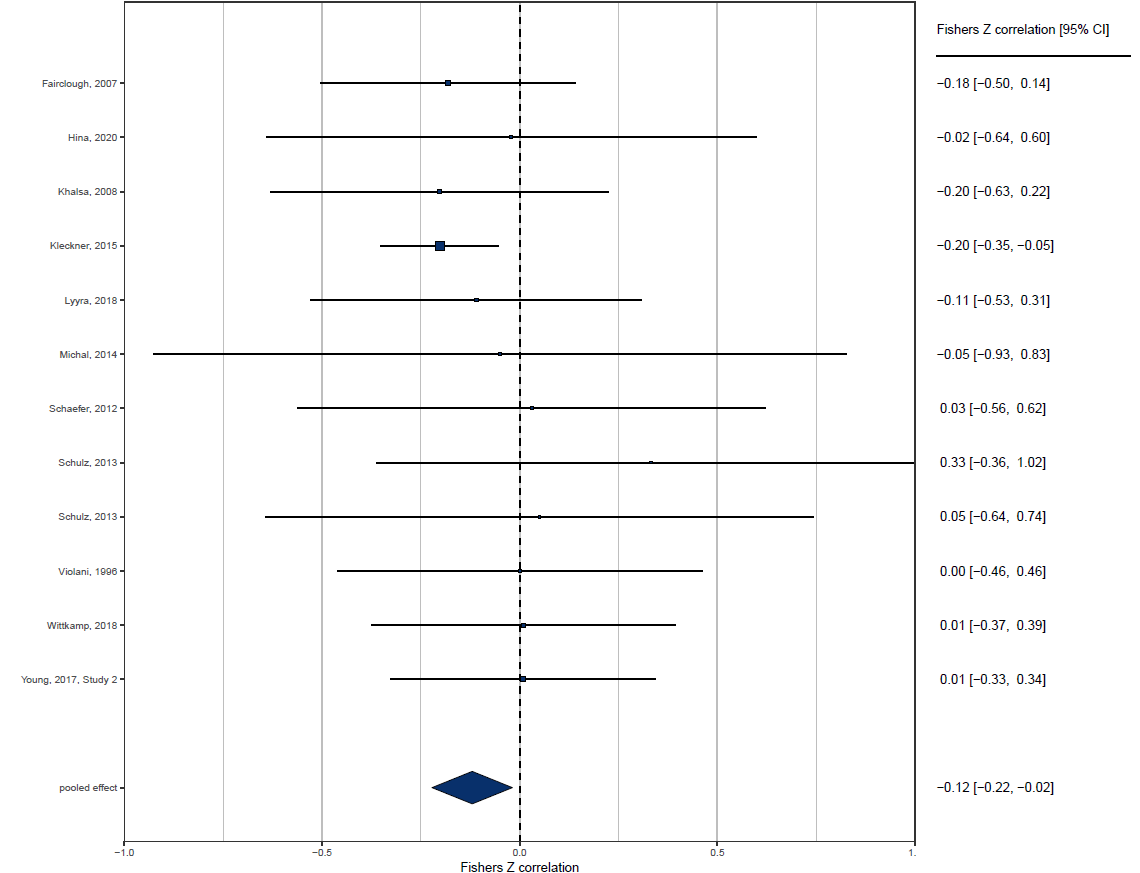


References

1. Jenkinson PM, Taylor L, Laws KR. Self-reported interoceptive deficits in eating disorders: A meta-analysis of studies using the eating disorder inventory. *Journal of Psychosomatic Research* 2018;110:38-45. doi: <https://doi.org/10.1016/j.jpsychores.2018.04.005>

2. Simmons JP, Nelson LD, Simonsohn U. False-positive psychology: undisclosed flexibility in data collection and analysis allows presenting anything as significant. *Psychological science* 2011;22(11):1359-66. doi: 10.1177/0956797611417632

3. Robinson E, Bevelander KE, Field M, et al. Methodological and reporting quality in laboratory studies of human eating behavior. *Appetite* 2018; 130, 321-326

4. Viechtbauer W, Cheung MW-L. Outlier and influence diagnostics for meta-analysis. *Research Synthesis Methods* 2010;1(2):112-25. doi: 10.1002/jrsm.11

5. Egger M, Smith GD, Schneider M, et al. Bias in meta-analysis detected by a simple, graphical test. *BMJ* 1997;315(7109):629-34. doi: 10.1136/bmj.315.7109.629

6. Duval S, Tweedie R. Trim and fill: A simple funnel-plot-based method of testing and adjusting for publication bias in meta-analysis. *Biometrics* 2000;56(2):455-63. doi: 10.1111/j.0006-341x.2000.00455.x

7. Aaron RV, Blain SD, Snodgress MA, et al. Quadratic Relationship Between Alexithymia and Interoceptive Accuracy, and Results From a Pilot Mindfulness Intervention. *Frontiers in Psychiatry* 2020;11:132.

8. Abbate-Daga G, Buzzichelli S, Marzola E, et al. Clinical investigation of set-shifting subtypes in anorexia nervosa. *Psychiatry research* 2014;219(3):592-97. doi: 10.1016/j.psychres.2014.06.024

9. Ainley V, Tajadura-Jiménez A, Fotopoulou A, et al. Looking into myself: changes in interoceptive sensitivity during mirror self-observation. *Psychophysiology* 2012;49(11):1504-08. doi: 10.1111/j.1469-8986.2012.01468.

10. Ainley V, Tsakiris M. Body conscious? Interoceptive awareness, measured by heartbeat perception, is negatively correlated with self-objectification. *PloS one* 2013;8(2):e55568.

11. Ainley V, Brass M, Tsakiris M. Heartfelt imitation: high interoceptive awareness is linked to greater automatic imitation. *Neuropsychologia* 2014;60:21-28.

12. Ainley VL. The Heartfelt Self:Investigating Interactions between Individual Differences in Interoceptive Accuracy and Aspects of Self-Processing. *PhD thesis* 2015.

13. Amianto F, Bertorello A, Migliore F, et al. Alexithymia in anorexia and bulimia: Ubiquitous and primary trait? *Cogent Psychology* 2016;3(1):1185994.

14. Anderson LM. Does what you think you feel, impact what you actually eat? An examination of alexithymia, interoceptive awareness, and loss of control eating in young women. Unpublished Thesis. *ProQuest Information & Learning*, 2018.

15. Ardizzi M, Ambrosecchia M, Buratta L, et al. Interoception and positive symptoms in schizophrenia. *Frontiers in Human Neuroscience* 2016;10 doi: 10.3389/fnhum.2016.00379

16. Cano Sokoloff N, Eguiguren ML, Wargo K, et al. Bone parameters in relation to attitudes and feelings associated with disordered eating in oligo-amenorrheic athletes, eumenorrheic athletes, and nonathletes. *Int J Eat Disord* 2015;48(5):522-26. doi: 10.1002/eat.22405

17. Carcieri EM. The role of interoceptive awareness and self-efficacy in the relationship between negative affect and eating disorders in college women. Unpublished Thesis. ProQuest Information & Learning, 2014.

18. Cella S, Iannaccone M, Ascione R, et al. Body dissatisfaction, abnormal eating behaviours and eating disorder attitude in homo- and heterosexuals. *Eat Weight Disord* 2010;15(3):e180-e85. doi: 10.3275/6866

19. Crucianelli L, Krahé C, Jenkinson PM, et al. Interoceptive ingredients of body ownership: Affective touch and cardiac awareness in the rubber hand illusion. *Cortex* 2018;104:180-92.

20. Daubenmier J, Sze J, Kerr CE, et al. Follow your breath: Respiratory interoceptive accuracy in experienced meditators. *Psychophysiology* 2013;50(8):777-89. doi: 10.1111/psyp.12057

21. De Witte NAJ, Sütterlin S, Braet C, et al. Getting to the Heart of Emotion Regulation in Youth: The Role of Interoceptive Sensitivity, Heart Rate Variability, and Parental Psychopathology. *PloS one* 2016;11(10):e0164615-e15. doi: 10.1371/journal.pone.0164615

22. Dorard G, Khorramian-Pour M. Hyperphagie boulimique: Liens avec la personnalité et l’émotionnalité = Binge eating disorder: Links with personality and emotionality. *L'Encéphale: Revue de psychiatrie clinique biologique et thérapeutique* 2017;43(2):114-19. doi: 10.1016/j.encep.2016.05.005

23. Duschek S, Werner NS, Reyes del Paso GA, et al. The contributions of interoceptive awareness to cognitive and affective facets of body experience. *Journal of Individual Differences* 2015;36(2):110-18. doi: 10.1027/1614-0001/a000165

24. Duschek S, Montoro CI, Reyes Del Paso GA. Diminished Interoceptive Awareness in Fibromyalgia Syndrome. *Behav Med* 2017;43(2):100-07. doi: 10.1080/08964289.2015.1094442

25. Edwards DJ, Young H, Johnston R. The immediate effect of therapeutic touch and deep touch pressure on range of motion, interoceptive accuracy and heart rate variability: A randomized controlled trial with moderation analysis. *Frontiers in Integrative Neuroscience* 2018;12 doi: 10.3389/fnint.2018.00041

26. Ehlers A, Breuer P. Increased cardiac awareness in panic disorder. *J Abnorm Psychol* 1992;101(3):371.

27. Emanuelsen L, Drew R, Köteles F. Interoceptive sensitivity, body image dissatisfaction, and body awareness in healthy individuals. *Scandinavian Journal of Psychology* 2015;56(2):167-74. doi: 10.1111/sjop.12183

28. Engler PA, Crowther JH, Dalton G, et al. Predicting eating disorder group membership: An examination and extension of the sociocultural model. *Behav Ther* 2006;37(1):69-79.

29. Erle TM. Level-2 visuo-spatial perspective-taking and interoception–More evidence for the embodiment of perspective-taking. *PloS one* 2019;14(6):e0219005.

30. Fairclough SH, Goodwin L. The effect of psychological stress and relaxation on interoceptive accuracy: Implications for symptom perception. *Journal of Psychosomatic Research* 2007;62(3):289-95. doi: 10.1016/j.jpsychores.2006.10.017

31. Fassino S, Amianto F, Rocca G, et al. Parental bonding and eating psychopathology in bulimia nervosa: personality traits as possible mediators. *Epidemiology and Psychiatric Sciences* 2010;19(3):214-22.

32. Ferentzi E, Horváth Á, Köteles F. Do body-related sensations make feel us better? Subjective well-being is associated only with the subjective aspect of interoception. *Psychophysiology* 2019;56(4):e13319. doi: 10.1111/psyp.13319

33. Frank GKW, Shott ME, Keffler C, et al. Extremes of eating are associated with reduced neural taste discrimination. *Int J Eat Disord* 2016;49(6):603-12. doi: 10.1002/eat.22538

34. Furman DJ, Waugh CE, Bhattacharjee K, et al. Interoceptive awareness, positive affect, and decision making in major depressive disorder. *J Affect Disord* 2013;151(2):780-85.

35. García-Cordero I, Sedeño L, de la Fuente L, et al. Feeling, learning from and being aware of inner states: interoceptive dimensions in neurodegeneration and stroke. *Philos Trans R Soc Lond B Biol Sci* 2016;371(1708):20160006. doi: 10.1098/rstb.2016.0006

36. Golay A, Hagon I, Painot D, et al. Personalities and alimentary behaviors in obese patients. *Patient Educ Couns* 1997;31(2):103-12. doi: 10.1016/s0738-3991(97)00995-6

37. Herbert BM, Pollatos O. Attenuated interoceptive sensitivity in overweight and obese individuals. *Eating Behaviors* 2014;15(3):445-48. doi: 10.1016/j.eatbeh.2014.06.002

38. Hina F, Aspell J, Cardini F. Enhanced behavioural and brain responses to interoceptive signals in musicians. https://psyarxiv.com/smdwc/ 2020, pre-print.

39. Iorio D, Margiotta N, D'Orsi P, et al. The Eating Disorder Inventory in evaluation of impaired eating behaviour in subjects requesting nutritional consultation. *Eat Weight Disord* 2000;5(4):206-10. doi: 10.1007/bf03354447

40. Kaisari P, Dourish CT, Rotshtein P, et al. Associations between core symptoms of attention deficit hyperactivity disorder and both binge and restrictive eating. *Frontiers in Psychiatry* 2018;9(MAR) doi: 10.3389/fpsyt.2018.00103

41. Khalsa SS, Rudrauf D, Damasio AR, et al. Interoceptive awareness in experienced meditators. *Psychophysiology* 2008;45(4):671-77. doi: 10.1111/j.1469-8986.2008.00666.x

42. Khalsa SS, Rudrauf D, Tranel D. Interoceptive awareness declines with age. *Psychophysiology* 2009;46(6):1130-36. doi: 10.1111/j.1469-8986.2009.00859.x

43. Khalsa SS, Craske MG, Li W, et al. Altered interoceptive awareness in anorexia nervosa: effects of meal anticipation, consumption and bodily arousal. *International Journal of Eating Disorders* 2015;48(7):889-97.

44. Khalsa SS, Rudrauf D, Hassanpour MS, et al. The practice of meditation is not associated with improved interoceptive awareness of the heartbeat. *Psychophysiology* 2019 doi: 10.1111/psyp.13479

45. Kleckner IR, Wormwood JB, Simmons WK, et al. Methodological recommendations for a heartbeat detection-based measure of interoceptive sensitivity. *Psychophysiology* 2015;52(11):1432-40. doi: 10.1111/psyp.12503

46. Koch A, Pollatos O. Cardiac sensitivity in children: sex differences and its relationship to parameters of emotional processing. *Psychophysiology* 2014;51(9):932-41. doi: 10.1111/psyp.12233

47. Krautwurst S, Gerlach AL, Witthöft M. Interoception in pathological health anxiety. *J Abnorm Psychol* 2016;125(8):1179-84. doi: 10.1037/abn0000210

48. Kunstman JW, Clerkin EM, Palmer K, et al. The power within: The experimental manipulation of power interacts with trait BDD symptoms to predict interoceptive accuracy. *J Behav Ther Exp Psychiatry* 2016;50:178-86. doi: 10.1016/j.jbtep.2015.08.003

49. Lackner RJ, Fresco DM. Interaction effect of brooding rumination and interoceptive awareness on depression and anxiety symptoms. *Behav Res Ther* 2016;85:43-52. doi: 10.1016/j.brat.2016.08.007

50. Lewis DM, Cachelin FM. Body image, body dissatisfaction, and eating attitudes in midlife and elderly women. *Eating Disorders: The Journal of Treatment & Prevention* 2001;9(1):29-39. doi: 10.1080/106402601300187713

51. Limbrunner HM. Hyperfeminity, gender-role discrepancy, and eating disorders in varying high school environments. Unpublished Thesis. ProQuest Information & Learning, 2004.

52. Limmer J, Kornhuber J, Martin A. Panic and comorbid depression and their associations with stress reactivity, interoceptive awareness and interoceptive accuracy of various bioparameters. *J Affect Disord* 2015;185:170-79. doi: 10.1016/j.jad.2015.07.010

53. Lutz APC, Schulz A, Voderholzer U, et al. Enhanced cortical processing of cardio-afferent signals in anorexia nervosa. *Clinical Neurophysiology* 2019;130(9):1620-27. doi: 10.1016/j.clinph.2019.06.009

54. Lyyra P, Parviainen T. Behavioral inhibition underlies the link between interoceptive sensitivity and anxiety-related temperamental traits. *Frontiers in psychology* 2018;9:1026.

55. Maeda S, Ogishima H, Shimada H. Acute cortisol response to a psychosocial stressor is associated with heartbeat perception. *Physiol Behav* 2019;207:132-38.

56. Mata F, Verdejo-Roman J, Soriano-Mas C, et al. Insula tuning towards external eating versus interoceptive input in adolescents with overweight and obesity. *Appetite* 2015;93:24-30. doi: 10.1016/j.appet.2015.03.024

57. Michael GA, Naveteur J, Dupuy MA, et al. My heart is in my hands: The interoceptive nature of the spontaneous sensations felt on the hands. *Physiology and Behavior* 2015;143:113-20. doi: 10.1016/j.physbeh.2015.02.030

58. Michal M, Reuchlein B, Adler J, et al. Striking discrepancy of anomalous body experiences with normal interoceptive accuracy in depersonalization-derealization disorder. *PloS one* 2014;9(2):e89823-e23. doi: 10.1371/journal.pone.0089823

59. Mölbert SC, Sauer H, Dammann D, et al. Multimodal body representation of obese children and adolescents before and after weight-Loss treatment in comparison to normal-Weight children. *PLoS ONE* 2016;11(11) doi: 10.1371/journal.pone.0166826

60. Morrissey RA. Toward a more comprehensive understanding of bulimic-symptom expression in adolescent girls: Examining contingencies of the restraint pathway. Unpublised Thesis. ProQuest Information & Learning, 2018.

61. Muhtadie L. Emotional response coherence and interoceptive awareness: Development and validation of a novel assessment method. Unpublished Thesis. ProQuest Information & Learning, 2018.

62. Murphy J, Geary H, Millgate E, et al. Direct and indirect effects of age on interoceptive accuracy and awareness across the adult lifespan. *Psychonomic Bulletin and Review* 2018;25(3):1193-202. doi: 10.3758/s13423-017-1339-z

63. Murphy J, Cheesman R, Gregory AM, et al. Estimating the stability of heartbeat counting in middle childhood: A twin study. *Biol Psychol* 2019;148 doi: 10.1016/j.biopsycho.2019.107764

64. Murphy J, Brewer R, Plans D, et al. Testing the independence of self-reported interoceptive accuracy and attention. *Quarterly Journal of Experimental Psychology* 2020;73(1):115-33.

65. Otten S, Schötz E, Wittmann M, et al. Psychophysiology of duration estimation in experienced mindfulness meditators and matched controls. *Frontiers in psychology* 2015;6:1215.

66. Parra-Fernández M-L, Rodríguez-Cano T, Onieva-Zafra M-D, et al. Prevalence of orthorexia nervosa in university students and its relationship with psychopathological aspects of eating behaviour disorders. *BMC Psychiatry* 2018;18(1):364-64. doi: 10.1186/s12888-018-1943-0

67. Richard A, Meule A, Georgii C, et al. Associations between interoceptive sensitivity, intuitive eating, and body mass index in patients with anorexia nervosa and normal-weight controls. *European Eating Disorders Review* 2019;27(5):571-77. doi: 10.1002/erv.2676

68. Robinson E, Marty L, Higgs S, Jones, A. Interoception, eating behaviour and heavier body weight. *University of Liverpool unpublished manuscript* 2020

69. Schaefer M, Egloff B, Witthöft M. Is interoceptive awareness really altered in somatoform disorders? Testing competing theories with two paradigms of heartbeat perception. *J Abnorm Psychol* 2012;121(3):719.

70. Schäflein E, Sattel HC, Pollatos O, et al. Disconnected–impaired interoceptive accuracy and its association with self-perception and cardiac vagal tone in patients with dissociative disorder. *Frontiers in Psychology* 2018;9:897.

71. Schlinkert C, Herbert BM, Baumann N, et al. Preoccupied with the body: mild stress amplifies the relation between rumination and interoception. *Cognition and Emotion* 2020:1-13.

72. Schulz, A., Lass-Hennemann, J., Sütterlin, S., Schächinger, H., & Vögele, C. (2013). Cold pressor stress induces opposite effects on cardioceptive accuracy dependent on assessment paradigm. *Biological Psychology*, *93*(1), 167-174.

73. Sehm M, Warschburger P. The Specificity of Psychological Factors Associated with Binge Eating in Adolescent Boys and Girls. *J Abnorm Child Psychol* 2015;43(8):1563-71. doi: 10.1007/s10802-015-0026-7

74. Shah P, Hall R, Catmur C, et al. Alexithymia, not autism, is associated with impaired interoception. *Cortex* 2016;81:215-20.

75. Tabor A, Vollaard N, Keogh E, et al. Predicting the consequences of physical activity: An investigation into the relationship between anxiety sensitivity, interoceptive accuracy and action. *PloS one* 2019;14(3):e0210853-e53. doi: 10.1371/journal.pone.0210853

76. Tiggemann M, Williams E. The role of self-objectification in disordered eating, depressed mood, and sexual functioning among women: A comprehensive test of objectification theory. *Psychology of Women Quarterly* 2012;36(1):66-75. doi: 10.1177/0361684311420250

77. Todd J, Aspell JE, Barron D, et al. An exploration of the associations between facets of interoceptive awareness and body image in adolescents. *Body Image* 2019;31:171-80. doi: 10.1016/j.bodyim.2019.10.004

78. Todd J, Aspell JE, Barron D, et al. Multiple dimensions of interoceptive awareness are associated with facets of body image in British adults. *Body Image* 2019;29:6-16. doi: 10.1016/j.bodyim.2019.02.003

79. Todd J, Aspell JE, Barron D, et al. Greater gastric interoception is associated with more positive body image: Evidence from adults in Malaysia and the United Kingdom. *Body Image* 2020;34:101-11.

80. Tylka TL, Kroon Van Diest AM. The Intuitive Eating Scale–2: Item refinement and psychometric evaluation with college women and men. *J Couns Psychol* 2013;60(1):137-53. doi: 10.1037/a003089310.1037/a0030893.supp (Supplemental)

81. van Dyck Z, Herbert BM, Happ C, et al. German version of the Intuitive Eating Scale: Psychometric evaluation and application to an eating disordered population. *Appetite* 2016;105:798-807. doi: 10.1016/j.appet.2016.07.019

82. van Strien T. Ice-cream consumption, tendency toward overeating, and personality. *Int J Eat Disord* 2000;28(4):460-64.

83. Van Strien T, Engels RCME, Van Leeuwe J, et al. The Stice model of overeating: tests in clinical and non-clinical samples. *Appetite* 2005;45(3):205-13. doi: 10.1016/j.appet.2005.08.004

84. van Strien T, Ouwens MA. Effects of distress, alexithymia and impulsivity on eating. *Eating behaviors* 2007;8(2):251-57. doi: 10.1016/j.eatbeh.2006.06.004

85. Violani C, Lombardo C, De Gennaro L, et al. Left movers' advantage in heartbeat discrimination: A replication and extension. *Psychophysiology* 1996;33(3):234-38. doi: 10.1111/j.1469-8986.1996.tb00420.x

86. Willem C, Gandolphe M-C, Roussel M, et al. Difficulties in emotion regulation and deficits in interoceptive awareness in moderate and severe obesity. *Eat Weight Disord* 2019;24(4):633-44. doi: 10.1007/s40519-019-00738-0

87. Wittkamp MF, Bertsch K, Vögele C, et al. A latent state‐trait analysis of interoceptive accuracy. *Psychophysiology* 2018;55(6):1-14. doi: 10.1111/psyp.13055

88. Yoris A, Abrevaya S, Esteves S, et al. Multilevel convergence of interoceptive impairments in hypertension: New evidence of disrupted body–brain interactions. *Hum Brain Mapp* 2018;39(4):1563-81.

89. Young HA, Williams C, Pink AE, et al. Getting to the heart of the matter: Does aberrant interoceptive processing contribute towards emotional eating? *PLoS ONE* 2017;12(10) doi: 10.1371/journal.pone.0186312

90. Young HA, Gaylor CM, de Kerckhove D, et al. Interoceptive accuracy moderates the response to a glucose load: a test of the predictive coding framework. *Proceedings of the Royal Society B* 2019;286(1898):20190244.

91. Bernatova T, Svetlak M. Emotional and interoceptive awareness and its relationship to restriction in young women with eating disorders and healthy controls: A cascade from emotional to behavioral dysregulation. *Activitas Nervosa Superior* 2017;59(2):78-86.

92. Koch A, Pollatos O. Interoceptive sensitivity, body weight and eating behavior in children: A prospective study. *Frontiers in Psychology* 2014;5(SEP) doi: 10.3389/fpsyg.2014.01003

93. Sehm M, Warschburger P. Prospective Associations Between Binge Eating and Psychological Risk Factors in Adolescence. *J Clin Child Adolesc Psychol* 2018;47(5):770-84. doi: 10.1080/15374416.2016.1178124

1. All interoception measures are standardised so that higher scores = better interoception (applicable to all tables). M = mean, SD = standard deviation, pps = participants.EDI-IA = Eating disorders inventory, interoceptive awareness subscale. MAIA = Multidimensional Assessment of Interoceptive Awareness. Study effect sizes may differ slightly to figures due to rounding and/or conversion. [↑](#footnote-ref-2)
2. All interoception measures are standardised so that higher scores = better interoception (applicable to all tables). M = mean, SD = standard deviation, pps = participants.MAIA = Multidimensional Assessment of Interoceptive Awareness. [↑](#footnote-ref-3)
3. All interoception measures are standardised so that higher scores = better interoception (applicable to all tables). M = mean, SD = standard deviation, pps = participants.Mixed indicates measure is calculated from objective task performance and self-report.Study effect sizes may differ slightly to figures due to rounding and/or conversion. [↑](#footnote-ref-4)
4. All interoception measures are standardised so that higher scores = better interoception (applicable to all tables). M = mean, SD = standard deviation, pps = participants.EDI-IA = Eating disorders inventory, interoceptive awareness subscale.Study effect sizes may differ slightly to figures due to rounding and/or conversion. [↑](#footnote-ref-5)
5. Analysis adjusted indicates whether covariates were included in analysis. Diagnosis PC/ED excluded refers to whether participants with psychiatric conditions/eating disorders were removed from analyses. BMI variance indicates whether acceptable variability in participant BMI was present in sample. UW pps removed indicates whether participants with a BMI < 18.5 were removed from analyses. Measure problems indicates whether any potential issues with included interoception measure were noted. N = no, Y = yes, U = unclear. MAIA = Multidimensional Assessment of Interoceptive Awareness.EDI-IA = Eating disorders inventory, interoceptive awareness subscale. [↑](#footnote-ref-6)
6. Analysis adjusted indicates whether covariates were included in analysis. Diagnosis PC/ED excluded refers to whether participants with psychiatric conditions/eating disorders were removed from analyses. BMI variance indicates whether acceptable variability in participant BMI was present in sample. UW pps removed indicates whether participants with a BMI < 18.5 were removed from analyses. Measure problems indicates whether any potential issues with included interoception measure were noted. N = no, Y = yes, U = unclear. MAIA = Multidimensional Assessment of Interoceptive Awareness. [↑](#footnote-ref-7)
7. Analysis adjusted indicates whether covariates were included in analysis. Diagnosis PC/ED excluded refers to whether participants with psychiatric conditions/eating disorders were removed from analyses. BMI variance indicates whether acceptable variability in participant BMI was present in sample. UW pps removed indicates whether participants with a BMI < 18.5 were removed from analyses. Measure problems indicates whether any potential issues with included interoception measure were noted. N = no, Y = yes, U = unclear. Mixed refers to interoception measure being calculated using objective task performance and self-reported data. [↑](#footnote-ref-8)
8. Analysis adjusted indicates whether covariates were included in analysis. Diagnosis PC/ED excluded refers to whether participants with psychiatric conditions/eating disorders were removed from analyses. BMI variance indicates whether acceptable variability in participant BMI was present in sample. UW pps removed indicates whether participants with a BMI < 18.5 were removed from analyses. Measure problems indicates whether any potential issues with included interoception measure were noted. N = no, Y = yes, U = unclear. EDI-IA = Eating disorders inventory, interoceptive awareness subscale. [↑](#footnote-ref-9)
